# Cationic Cobalt(II) Bisphosphine Hydroformylation Catalysis: In Situ Spectroscopic and Reaction Studies

**DOI:** 10.1021/jacs.3c04866

**Published:** 2023-08-29

**Authors:** Drew M. Hood, Ryan A. Johnson, David J. Vinyard, Frank R. Fronczek, George G. Stanley

**Affiliations:** † Department of Chemistry, Louisiana State University, Baton Rouge, Louisiana 70803-1804, United States; ‡ Department of Biological Sciences, Louisiana State University, Baton Rouge, Louisiana 70803-1804, United States

## Abstract

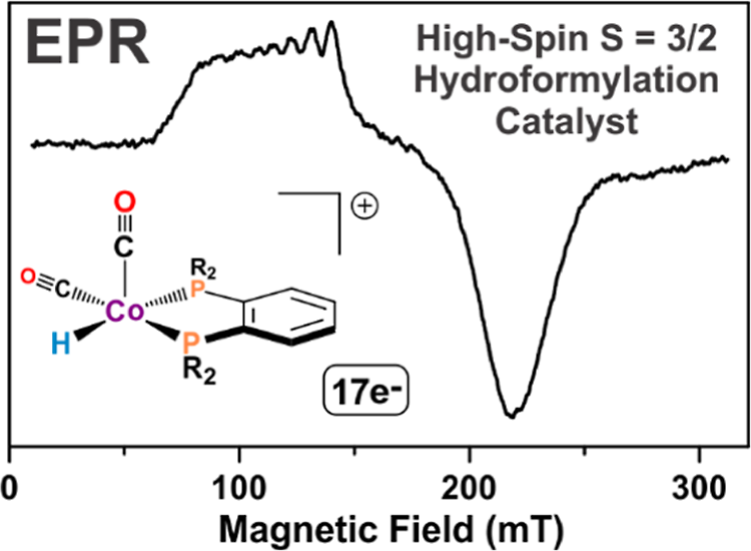

[HCo(CO)_*x*_(bisphosphine)](BF_4_), *x* = 1–3, is a highly active hydroformylation catalyst system, especially for internal branched alkenes. In situ infrared spectroscopy (IR), electron paramagnetic resonance (EPR), and nuclear magnetic resonance studies support the proposed catalyst formulation. IR studies reveal the formation of a dicationic Co(I) paramagnetic CO-bridged dimer, [Co_2_(μ-CO)_2_(CO)(bisphosphine)_2_]^2+^, at lower temperatures formed from the reaction of two catalyst complexes via the elimination of H_2_. DFT studies indicate a dimer structure with square-pyramidal and tetrahedral cobalt centers. This monomer–dimer equilibrium is analogous to that seen for HCo(CO)_4_, reacting to eliminate H_2_ and form Co_2_(CO)_8_. EPR studies on the catalyst show a high-spin (*S* = 3/2) Co(II) complex. Reaction studies are presented that support the cationic Co(II) bisphosphine catalyst as the catalyst species present in this system and minimize the possible role of neutral Co(I) species, HCo(CO)_4_ or HCo(CO)_3_(phosphine), as catalysts.

## Introduction

**Scheme 1:**
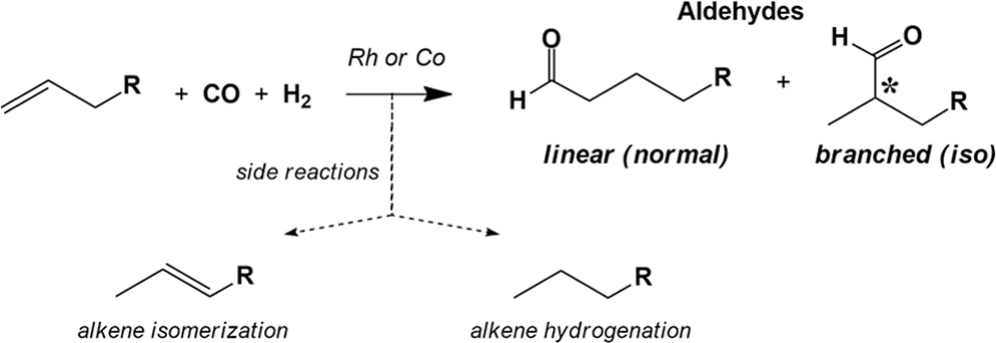
Hydroformylation.

Cobalt was the first metal discovered to catalyze hydroformylation, or the oxo reaction (Scheme 1), by Otto Roelen in 1938 while studying Fischer–Tropsch catalysis.^1^ Heck proposed in 1960 that HCo(CO)_4_ was the catalyst species responsible for hydroformylation, along with the currently accepted catalytic mechanism.^2,3^ Although HCo(CO)_4_ is highly active, one significant problem is that it readily decomposes to cobalt metal as the temperature is increased unless enough CO partial pressure is present. Industrial processes based on HCo(CO)_4_ typically run around 180 °C and use H_2_/CO pressures over 200 bar, which is considered a high-pressure process.

Slaugh and Mullineaux at Shell Chemical discovered in the late 1960s that adding an electron-donating, sterically bulky, alkylated phosphine ligand to the cobalt(I) center generated the phosphine-modified catalyst system, HCo(CO)_3_(PR_3_).^4,5^ This catalyst had two significant advantages over the unmodified HCo(CO)_4_ catalyst: (1) increased stability toward decomposition to cobalt metal that allowed considerably lower CO partial pressures (typically 25–30 bar) even at higher temperatures and (2) significantly increased aldehyde linear/branched (L/B) ratios, typically around 8:1 L/B. The increased L/B aldehyde ratio was particularly important to Shell because they hydroformylate internal linear alkenes generated from the Shell higher olefin process (i.e., oligomerization of ethylene). The phosphine-modified HCo(CO)_3_(PR_3_) catalyst had high alkene isomerization properties, similar to HCo(CO)_4_, but could be run under medium pressure industrial conditions.

There are, however, several downsides to the phosphine-modified cobalt catalyst system. The increased stability toward decomposition to cobalt metal is due to the σ-donation of the phosphine ligand, which increased the electron-density of the cobalt center, resulting in stronger π-backbonding to the carbonyl ligands. This dramatically lowered the activity of the catalyst that is dependent on CO dissociation for reaction with alkenes and H_2_. Higher temperatures (typically around 190 °C) and increased catalyst loadings were needed to compensate for the lower catalyst activity. The increased phosphine ligand σ-donation also increases the hydricity of the catalyst, which enhances its hydrogenation activity. This hydrogenates the aldehyde to produce alcohol (desired), but also hydrogenates the alkene reactant to make alkane (highly undesired). The final problem for the phosphine-modified cobalt catalyst is that there is some phosphine ligand dissociation, especially at the higher temperatures used industrially. This generates the HCo(CO)_4_ catalyst that can decompose to cobalt metal.

Beller and coworkers recently reported that Co(I) hydroformylation could operate under milder conditions (20–40 bar, 60–120 °C) through the use of phosphine-oxide promoters.^6^ This system basically behaves like a HCo(CO)_4_-stabilized catalyst, but is quite slow with limited turnovers. They ran their experiments with a 3:1 H_2_/CO gas ratio, which favors higher initial turnover frequencies, but in a batch autoclave experiment, it will lead to CO depletion for extended catalytic runs.

**Figure 1:**
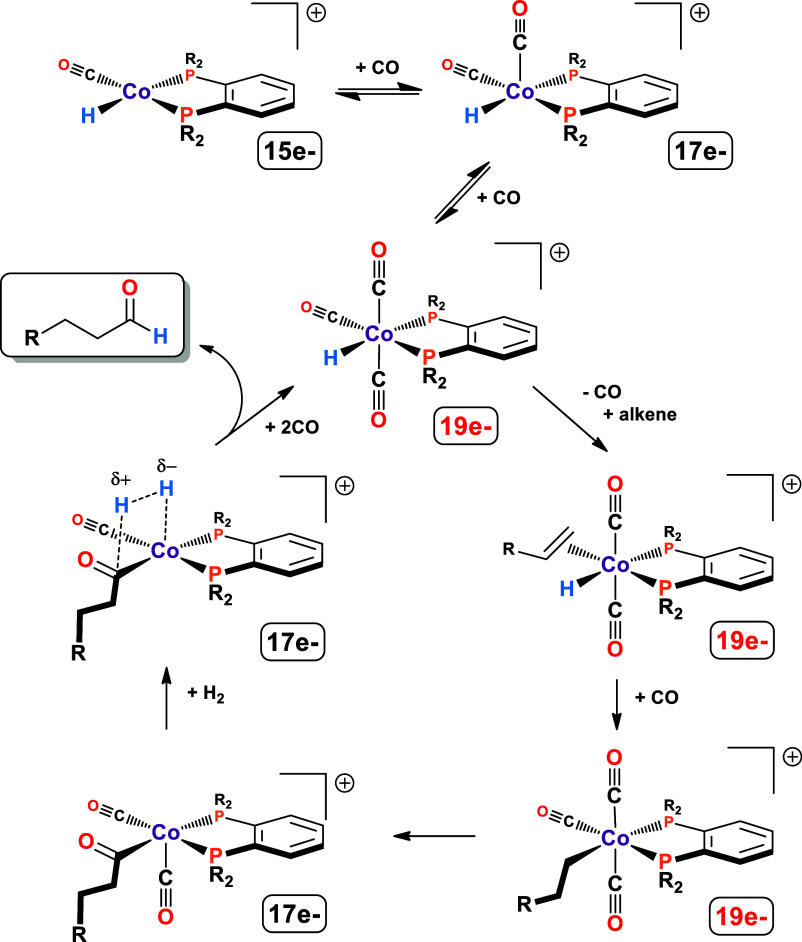
Proposed hydroformylation mechanism for the cationic Co(II) bisphosphine catalyst system. Reprinted with permission from ref (7).

These neutral Co(I) catalyst systems were the only ones known until 2020 when we, in conjunction with researchers at ExxonMobil, reported a cationic Co(II) chelating bisphosphine catalyst system, [HCo(CO)_*x*_(bisphosphine)](BF_4_), *x* = 1–3, which is active to highly active under a wide range of temperatures and pressures.^7^ The activity, for example, is only about 20 times slower than the best rhodium–phosphine catalysts for a nonisomerizable alkene such as *t*-butylethylene. This cationic Co(II) catalyst behaves in many ways similar to HCo(CO)_4_ but with considerably higher catalyst stability, allowing it to access temperatures and pressures at which HCo(CO)_4_ is unstable. The cationic Co(II) catalyst also has high alkene isomerization ability analogous to the neutral Co(I) catalysts. The proposed hydroformylation mechanism is shown in Figure 1.^7^

Similar to the phosphine-modified cobalt catalyst, it appears to have somewhat higher hydricity compared to HCo(CO)_4_ due to the donating bisphosphine ligand that allows it to hydrogenate aldehyde to alcohol when the aldehyde concentration builds up enough, but not hydridic enough to do much hydrogenation of alkene.

**Scheme 2:**
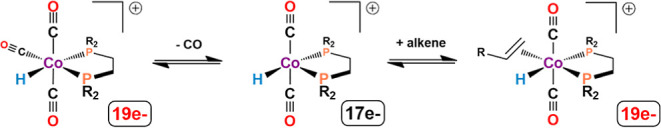
Alkene coordination to equatorial site.

The other significant difference versus the phosphine-modified cobalt(I) catalyst system is that the cationic Co(II) system generally has low L/B aldehyde regioselectivity (1:1 to 2:1 L/B) when dealing with 1-alkenes like 1-hexene, at least with the limited number of chelating phosphines studied by our research group.^7^ The reason for this was proposed to be that the primary alkene coordination site is trans to the chelating bisphosphine (cis to the hydride), as shown in Scheme 2, where it is least affected by the bisphosphine R-groups that are oriented away from the equatorial region. The axial coordination sites are too crowded to allow coordination of sterically hindered alkenes.

The equatorial CO coordination site, however, has the strongest Co–CO bond and is thus least likely to dissociate. The 19e^–^ tricarbonyl was proposed to play an important role, along with the localized cationic charge on the cobalt center, in helping labilize the equatorial carbonyl and allowing coordination of the alkene to initiate hydroformylation. Evidence for the 19e^–^ tricarbonyl catalyst came from in situ FT-IR studies that show a high frequency carbonyl band at 2086 cm^–1^ for the [HCo(CO)_3_(DPPBz)](BF_4_) catalyst (DPPBz = (Ph_2_P)_2_-1,2-C_6_H_4_).^7^

The other data supporting the role of the 19e^–^ tricarbonyl species comes from the fact that a higher catalyst activity is seen for the cationic Co(II) catalyst with electron-donating bisphosphine ligands such as Et_2_PCH_2_CH_2_PEt_2_ (depe) and (Et_2_P)_2_-1,2-C_6_H_4_ (DEPBz). There are no examples of either Co(I) or Rh(I) hydroformylation catalysts having higher activity with electron-donating phosphine ligands. More electron-donating phosphines favor the 19e^–^ tricarbonyl complex, [HCo(CO)_3_(bisphosphine)]^+^, which in turn helps labilize the more strongly coordinated equatorial CO ligand—the coordination site preferred by alkenes.

Franke and Zhang recently reported studies on HCo(CO)_4_ hydroformylation catalysis in which they report stability of this catalyst under medium pressure conditions and temperatures (e.g., 20 bar 1:1 H_2_/CO, 140 °C).^8^ The higher temperature/pressure sensitivity of HCo(CO)_4_ was shown by decomposition occurring at 20 bar and 160 °C. Additionally, they claimed to have prepared the cationic Co(II) catalyst precursor, [Co(acac)(DPPBz)](BF_4_), (acac = acetoacetonate)] and reported that it had low activity. They, furthermore, suggested that HCo(CO)_4_ was likely the true catalyst in the cationic Co(II) bisphosphine catalyst system.

Reported herein are additional in situ infrared spectroscopy (IR), nuclear magnetic resonance (NMR), and electron paramagnetic resonance (EPR) studies of the cationic Co(II) bisphosphine catalyst system. Reaction studies to probe the possible role of HCo(CO)_4_ as a significant component were performed and, along with the spectroscopic studies, demonstrate that [HCo(CO)_*x*_(bisphosphine)]^+^, *x* = 1–3, is the dominant hydroformylation catalyst system.

## Results and Discussion

### In Situ FT-IR Studies

**Figure 2:**
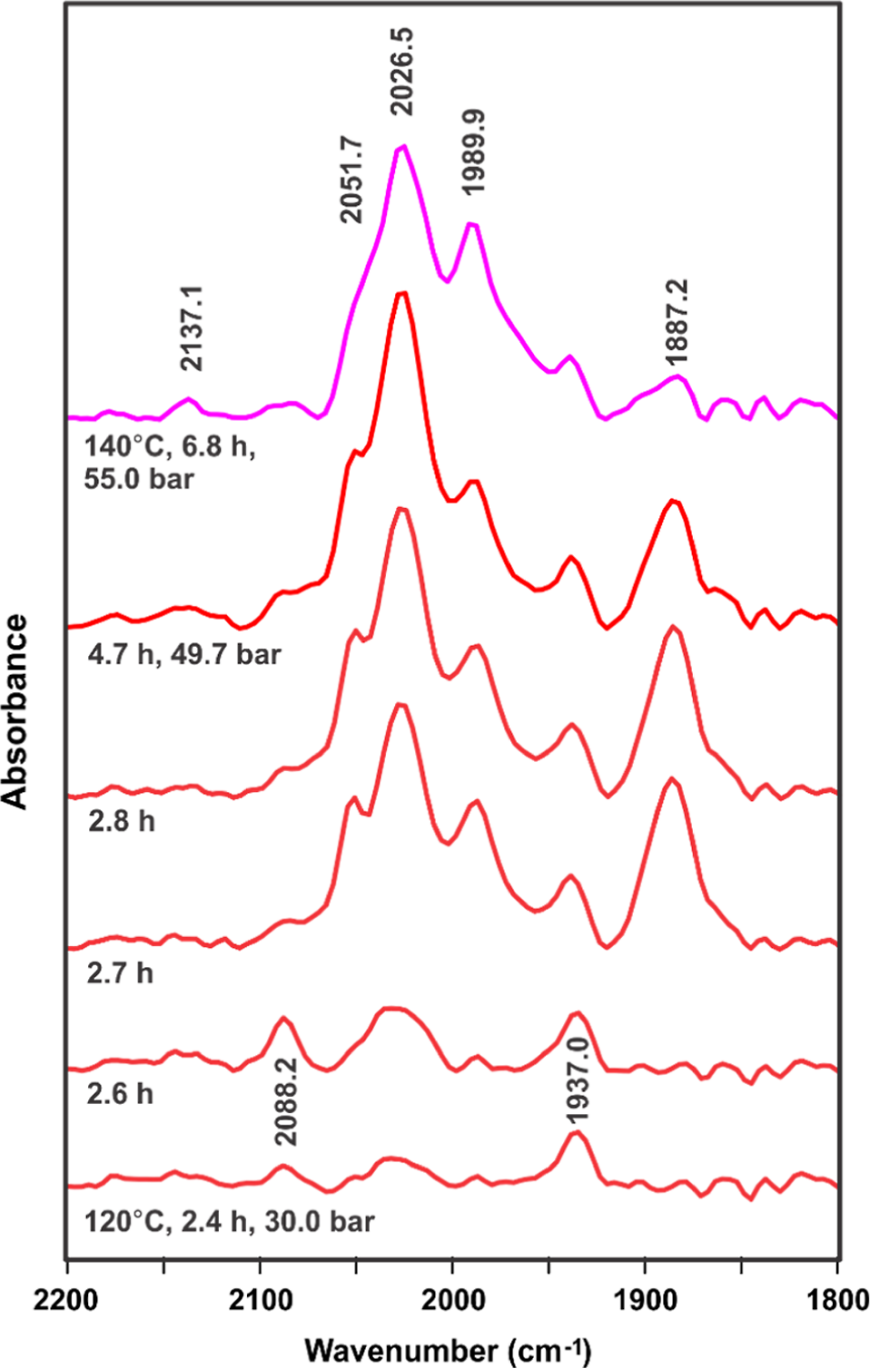
In situ FT-IR solvent-subtracted spectra using a high-pressure ReactIR system of the catalyst precursor, [Co(acac)(DPPBz)](BF_4_), under 1:1 H_2_/CO pressure at various temperatures in dimethoxytetraglyme solvent over the first 6.8 h. The band at 2137 cm^–1^ is due to dissolved CO in the solution.

**Figure 3:**
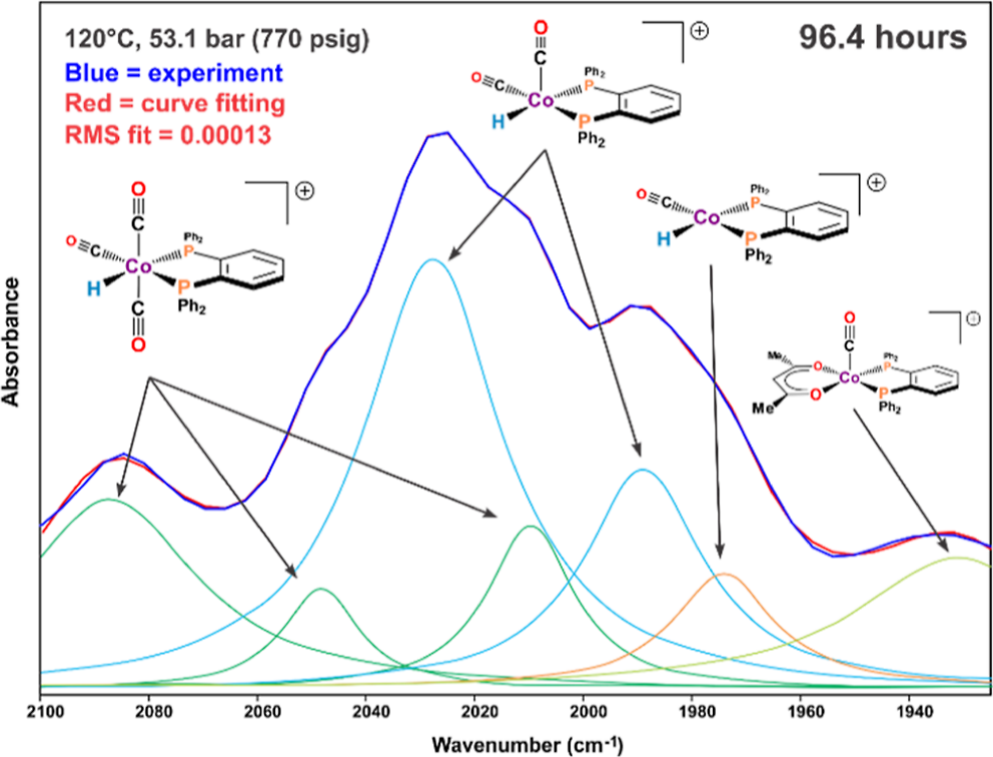
FT-IR curve fitting using a minimum number of Lorentzian peaks. Peak assignments (cm^–1^): [HCo(CO)_3_(DPPBz)]^+^ = 2085, 2049, and 2010; [HCo(CO)_2_(DPPBz)]^+^ = 2028 and 1989; [HCo(CO)(DPPBz)]^+^ = 1974 cm^–1^; [Co(acac)(CO)(DPPBz)]^+^ = 1938 cm^–1^. Reprinted with permission from ref (7).

**Figure 4:**
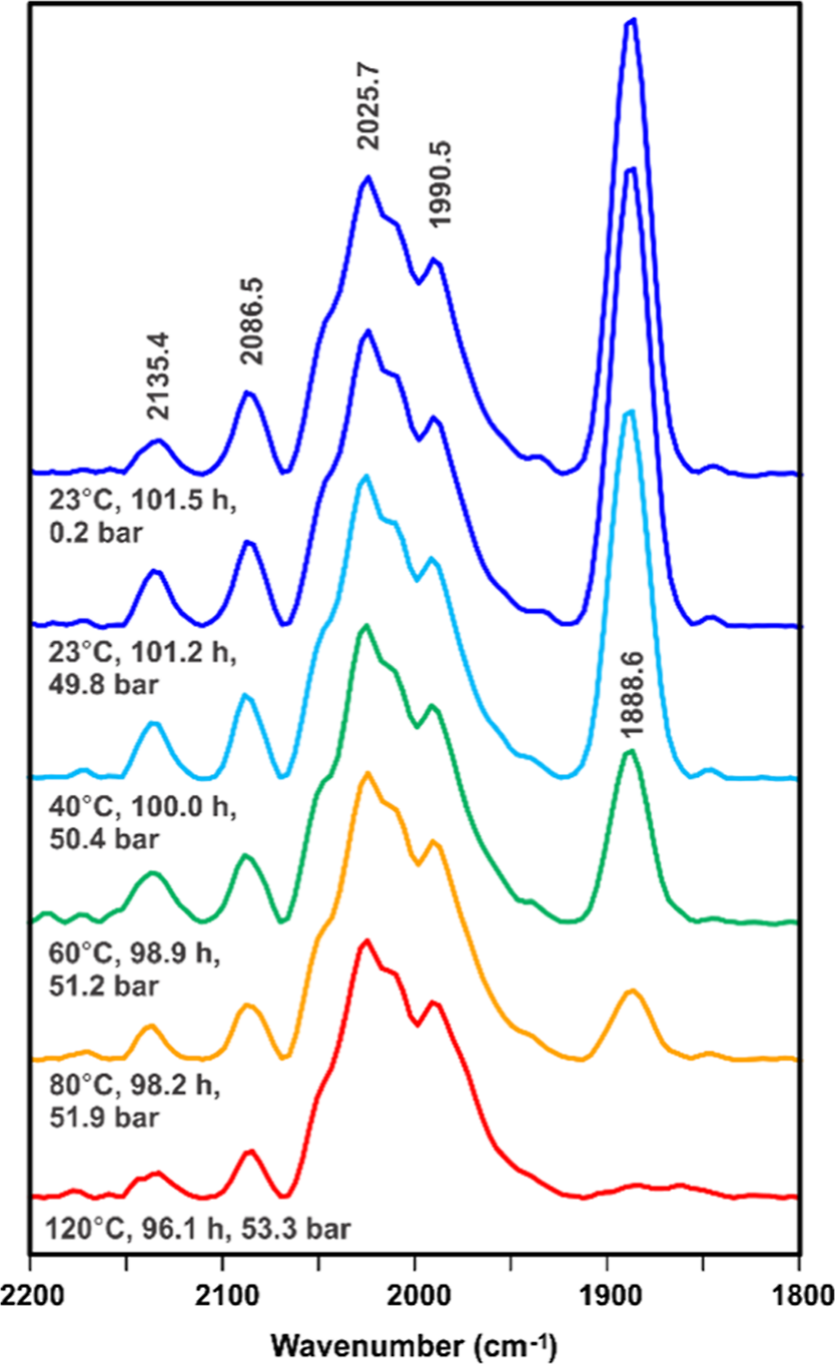
In situ FT-IR spectra of the catalyst system during the last 5 h of the experiment. Spectrum colors indicate temperature.

A number of in situ FT-IR studies of the cationic cobalt(II) bisphosphine catalyst system have been performed. Perhaps the most significant was a 101.5-h study probing the effect of different temperatures (generally around 30–54 bar 1:1 H_2_/CO), using the [Co(acac)(DPPBz)](BF_4_) catalyst precursor (10 mM concentration, dimethoxytetraglyme solvent). A high-pressure Mettler–Toledo ReactIR system with a silicon-windowed probe was used. A selection of the FT-IR spectra is shown in Figures 2–4 (full set in the Supporting Information).

The initial FT-IR spectra shown in Figure 2 show only a low-intensity cobalt carbonyl band at 1937 cm^–1^ representing the 5-coordinate, 17e^–^ complex [Co(acac)(CO)(DPPBz)]^+^. This appears at room temperature (10–30 bar) and stays about the same as the temperature is increased to 120 °C over the course of 2.5 h. The catalyst precursor starts to activate and react with H_2_ fairly quickly once the temperature reaches 120 °C.

A heterolytic reaction of H_2_ and [Co(acac)(CO)(DPPBz)]^+^ is proposed to dissociate acetylacetone (Hacac) and form the cationic Co(II) hydride–carbonyl catalyst system, [HCo(CO)_*x*_(DPPBz)]^+^, *x* = 1–3. The equilibrium mixture of the various carbonyl complexes has carbonyl bands ranging from 2088 to 1974 cm^–1^, with major carbonyl bands at 2027–2025 and 1990 cm^–1^. The 1937 cm^–1^ band representing the carbonyl-coordinated catalyst precursor, [Co(acac)(CO)(DPPBz)]^+^, gradually decreases over time.

Most surprising was the formation of a strong carbonyl band around 1888 cm^–1^ during the initial formation of the [HCo(CO)_*x*_(DPPBz)]^+^, *x* = 1–3, catalyst mixture. [Co(CO)_4_]^−^ anion has a strong carbonyl band^9^ at 1888 cm^–1^, and if this carbonyl band was due to [Co(CO)_4_]^−^ anion, it would indicate that the cationic Co(II) catalyst was falling apart to generate a mixture of HCo(CO)_4_ and [Co(CO)_4_]^−^ anion. As will be discussed later, the 1888 cm^–1^ carbonyl band is proposed to be due to a dicationic CO-bridged cobalt(I) dimer and not [Co(CO)_4_]^−^ anion.

Continued heating of the catalyst at 120 °C, however, caused the carbonyl band at 1888 cm^–1^ to decrease in intensity. Raising the temperature to 140 °C and pressure to 54.2 bar (1:1 H_2_/CO) caused an even quicker decrease in the 1888 cm^–1^ band. Lowering the temperature back to room temperature (23 °C) reforms the 1888 cm^–1^ peak and a similar set of carbonyl bands representing the catalyst complexes between 2088 and 1974 cm^–1^. The complex representing the 1888 cm^–1^ band is favored at lower temperatures and strongly disfavored at 120–140 °C.

Several heating and cooling cycles were performed over this extended in situ IR study (Supporting Information). Cooling from 120 to 140 °C to room temperature causes the 1888 cm^–1^ band to reappear. Heating at room temperature and increasing the temperature to 120–140 °C causes the 1888 cm^–1^ band to disappear.

A stability study at 120 °C and 53 bar (1:1 H_2_/CO) was performed between 33 and 96.4 h (Supporting Information).^7^ The 1888 cm^–1^ band completely disappears, and the bands proposed to be due to the [HCo(CO)_*x*_(DPPBz)]^+^, *x* = 1–3, catalyst system stay essentially the same. No change in the intensity of the IR was observed during this period, which indicates that there was no decomposition to cobalt metal.

Curve fitting of the 96.4 h IR carbonyl region, shown in Figure 3, allowed for assignment of carbonyl bands to the various carbonyl containing catalyst species. These are consistent with the number of carbonyl bands expected for each species as well as the shifting to higher frequencies for the complexes with higher numbers of carbonyl ligands. They also agree qualitatively with the DFT-predicted carbonyl frequencies.^7^

Figure 4 shows the last 5 h of the 101-h IR study in which cooling of the solution to room temperature and ambient pressure with the growth of the strong 1888 cm^–1^ band was noticed. Note the increased intensity of the 2086 cm^–1^ band at room temperature, which is assigned to the 19e^–^ tricarbonyl complex, [HCo(CO)_3_(DPPBz)]^+^. The solution from the ReactIR high-pressure cell was carefully transferred to a Schlenk flask under N_2_. The catalyst solution was then diluted to 1 mM with dimethoxytetraglyme and placed into an autoclave where a hydroformylation run with 1-hexene (1 M) was performed. The same catalytic results were observed compared to that seen with a fresh [Co(acac)(DPPBz)](BF_4_) catalyst precursor.

The extended in situ FT-IR study demonstrates the remarkable stability of the cationic [HCo(CO)_*x*_(DPPBz)]^+^, *x* = 1–3, catalyst mixture. Rhodium–phosphine-based hydroformylation catalysts are well known to attack and degrade phosphine ligands that have P–Ph, P–CH_2_Ph, or P–OR groups.^10−12^ This is an especially serious problem under operating conditions without any alkene present. Almost all active rhodium phosphine hydroformylation catalysts based on phosphine ligands with P–Ph, P–CH_2_Ph or P–OR groups will completely deactivate within 24 h under operating conditions without any alkene present. Furthermore, all Rh(I) phosphine catalysts require excess phosphine ligand to be present due to the facile rhodium–phosphine dissociative equilibrium. This is true even for most chelating bisphosphine ligands.

No excess chelating phosphine ligand is used in the cationic Co(II) catalyst system and essentially no bisphosphine ligand dissociative equilibrium problems were observed under normal catalytic conditions. A 1.2 million turnover hydroformylation study, for example, over a 2 week (336 h) period using 1-hexene and the cationic Co-DPPBz catalyst (6 M 1-hexene, 3 μM catalyst, 50 bar, 160 °C) showed no sign of catalyst decomposition.^7^ The lower activity of cobalt relative to rhodium appears to inhibit metal-induced phosphine ligand fragmentation reactions, even for the considerably more active cationic Co(II) catalyst system.

### Cobalt Dimer Formation

**Scheme 3:**
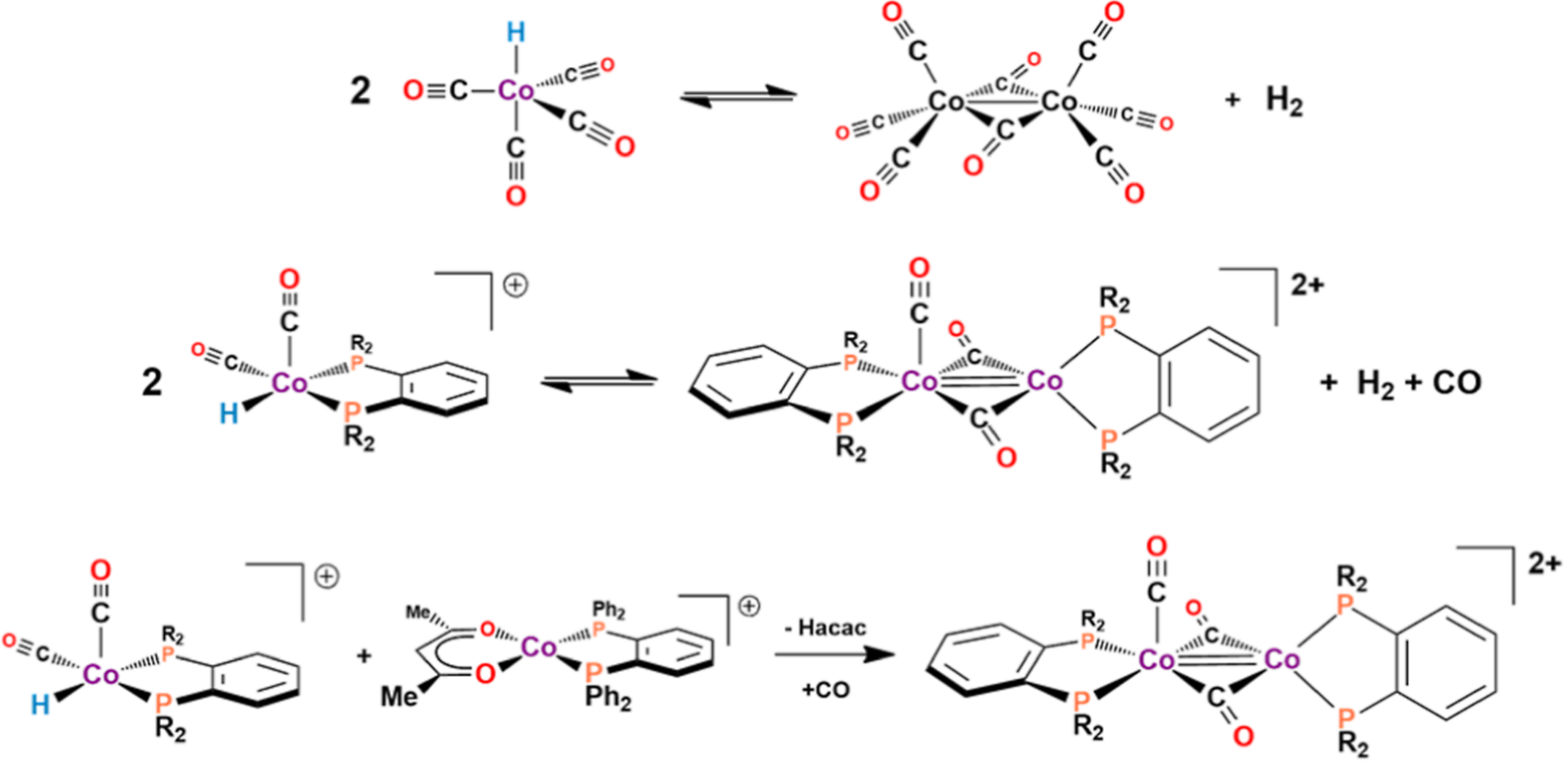
Cobalt dimer formation.

The 1888 cm^–1^ carbonyl band that grows in the in situ FT-IR at lower temperatures is proposed to be the dicationic Co(I) dimer, [Co_2_(μ-CO)_2_(CO)(DPPBz)_2_]^2+^ (Scheme 3). The reaction of two hydride catalysts, [HCo(CO)_*x*_(DPPBz)]^+^, *x* = 1–2, to reductively eliminate H_2_ forms the dicationic Co(I) carbonyl-bridged dimer. The dimer can oxidatively add H_2_ to reform two monometallic catalysts. This reaction is analogous to the equilibrium between two HCo(CO)_4_ monomers via the reductive elimination of H_2_ to produce Co_2_(CO)_8_ (Scheme 3 top).^13,14^

The proposed structure of the dicationic Co(I) dimer, [Co_2_(μ-CO)_2_(CO)(DPPBz)_2_]^2+^ (Scheme 3 middle), is based on DFT calculations (Supporting Information) and the fact that no ^31^P NMR is observed for this species which includes a high-spin (*S* = 1) Co(I) center. Although the square-pyramidal cobalt(I) center is expected to be formally diamagnetic, the close proximity and coupling to the high-spin tetrahedral Co(I) center makes the entire complex paramagnetic. The dimer is proposed to also form via the reaction of the catalyst [HCo(CO)_*x*_(bisphosphine)]^+^, *x* = 1–2, with the catalyst precursor [Co(acac)(bisphosphine)]^+^ via loss of Hacac (Scheme 3 bottom). The DFT study has a short Co–Co distance of 2.41 Å, which is consistent with a Co–Co double bond. Electron-counting with a Co–Co double bond gives 18e^–^ square-pyramidal and 16e^–^ tetrahedral Co(I) centers.

Figure 2 illustrates that as the catalyst precursor starts to react with H_2_ to form the catalyst, the 1888 cm^–1^ band due to dimer also forms at the same time. During the initial formation of [HCo(CO)_2_(bisphosphine)]^+^, the concentration of the catalyst precursor, [Co(acac)(bisphosphine)]^+^, is quite high and can react with the catalyst to directly form the dimer. The 120 °C temperature in Figure 2 during activation favors reaction of the dimer with H_2_ to form the catalyst complex. The higher concentration of the cobalt complex in the IR study (10 mM) compared to the catalytic runs (typically 1 mM) certainly could favor the dimer complex at lower temperatures. Catalysis, however, is typically done at temperatures where the dimer is unstable (>100 °C) and unlikely to play much of a role.

Kaim and coworkers reported^15^ the cationic Co(II) hydride bisphosphine complex [HCo(CO)_2_(dippf)]^+^ [dippf = 1,1′-bis(disopropylphosphino)ferrocene], which was generated electrochemically, has ν_CO_ = 2051, 2024 cm^–1^, very similar to what we observe, and reacts with itself to lose H_2_ and form the Co(I) cationic monomer [Co(CO)_2_(dippf)]^+^. The dippf bisphosphinoferrocene ligand does not have the right bite angle and sterics to allow the formation of an observable cobalt dimer.

The [Co_2_(μ-CO)_2_(CO)(DPPBz)_2_]^2+^ dimer is favored at lower temperatures, room temperature being the lowest studied, but the catalyst–dimer equilibrium under H_2_/CO still clearly shows a monomeric catalyst even at room temperature. The one terminal carbonyl on the dimer is proposed to be on the low-spin 5-coordinate Co(I) center and has a frequency around 2025 cm^–1^, overlapping with the carbonyl bands associated with the catalyst.

### EPR Studies

**Figure 5:**
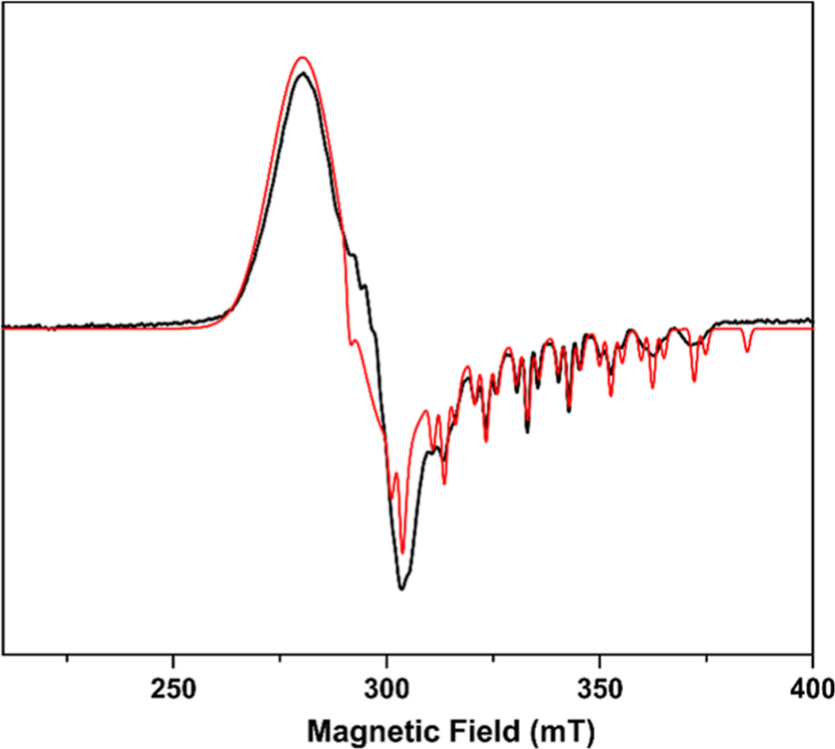
EPR spectra of [Co(acac)(DPPBz)](BF_4_) catalyst precursor at 5.5 K in 2-methyl-THF. Simulations in red. Reprinted with permission from ref (7).

The EPR spectrum of the [Co(acac)(DPPBz)](BF_4_) catalyst precursor is shown in Figure 5.^7^ The EPR spectrum is nearly axial with *g* = [2.41, 2.29, 2.01], consistent with low-spin (*S* = 1/2) Co(II). Hyperfine interactions from the *I* = 7/2 ^59^Co (100%) were simulated using principal values of 0, 0, and 275 MHz. Hyperfine interactions from two equivalent *I* = 1/2 ^31^P (100%) were simulated using principal values of 0, 0, and 350 MHz. Anisotropic line broadening was simulated^16^ using the H-strain tensor [500, 520, 30] MHz to account for unresolved hyperfine interactions. No other paramagnetic species were observed in the EPR. The EPR is completely consistent with the X-ray structure of the tetrahydrofuran (THF)-coordinated complex with square-pyramidal geometry.^7^

**Figure 6:**
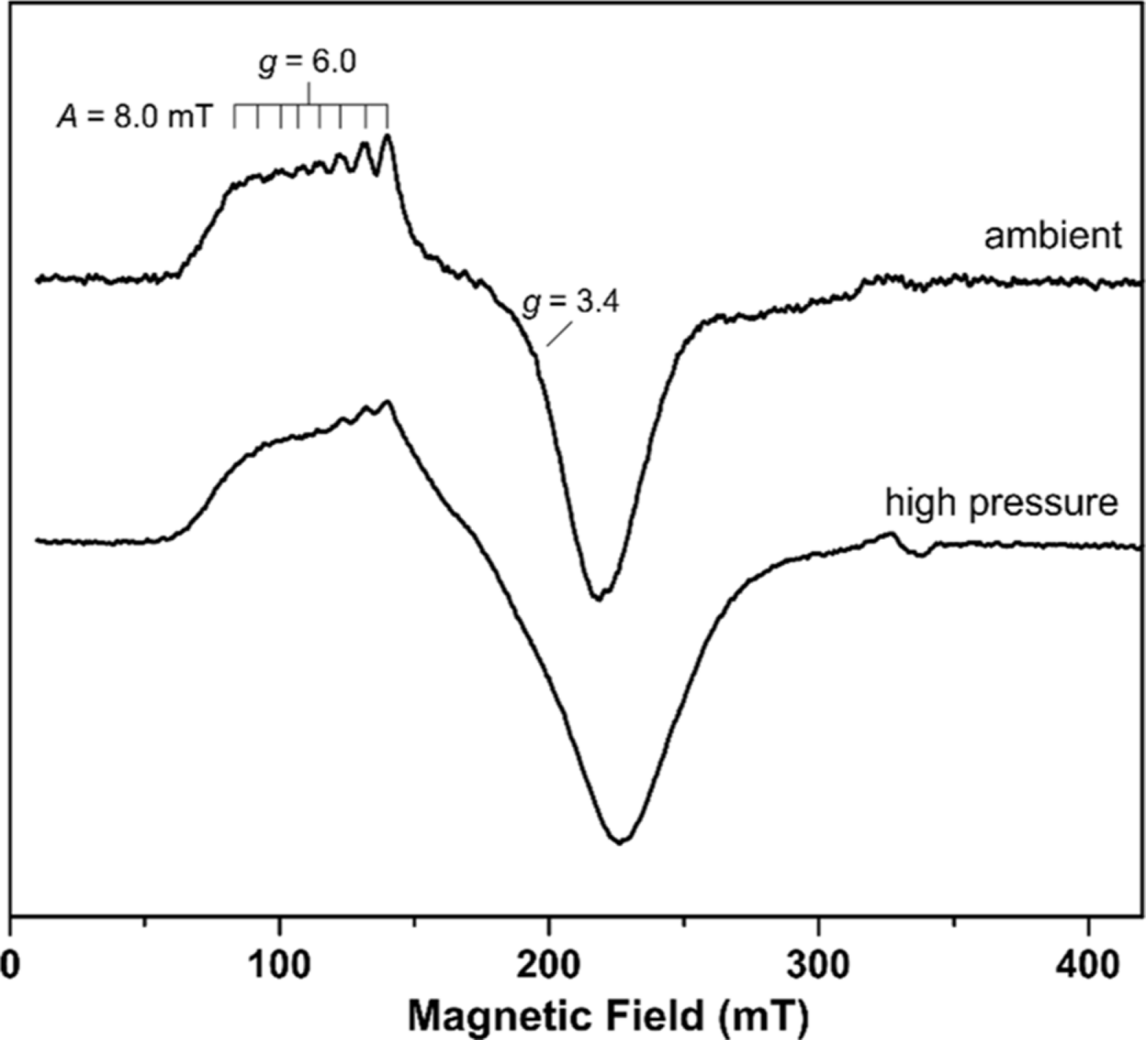
EPR spectra of [HCo(CO)_2_(DPPBz)](BF_4_) catalyst in 2-methyl-THF. (Top) Catalyst precursor activated in an autoclave, then transferred under ambient conditions to the EPR tube and cooled to 7 K. (Bottom) Catalyst precursor activated in a high-pressure quartz EPR tube under 27 bar H_2_/CO at 140 °C, then cooled to 6.7 K.

Two different EPR experiments were performed on the catalyst. The [Co(acac)(DPPBz)](BF_4_) catalyst precursor was activated in an autoclave under 30 bar of H_2_/CO at 140 °C using dimethoxytetraglyme solvent. The autoclave was cooled and depressurized, and a sample was withdrawn with a syringe, diluted with 2-methyl-THF (glass forming solvent), and transferred into a quartz EPR tube under an inert atmosphere. The sample was frozen in liquid nitrogen before being transferred to a helium cryostat in a Bruker EMX spectrometer and run at 7 K. The spectrum is shown in Figure 6 (top). The recorded EPR spectrum is rhombic with observed *g* values of approximately 6.0 and 3.4 consistent with high-spin (*S* = 3/2) Co(II). Hyperfine interactions from the *I* = 7/2 ^59^Co (100%) nucleus were estimated on the low field feature with *A* = 8.0 mT (670 MHz).

A second EPR experiment used a high-pressure quartz EPR tube that was loaded with [Co(acac)(DPPBz)](BF_4_) catalyst precursor dissolved in 2-methyl-THF, pressurized with 27 bar of H_2_/CO, and heated to 140 °C to activate the catalyst. It was then cooled to room temperature, followed by further cooling to 6.7 K for the EPR study. The high-pressure EPR spectrum is shown in Figure 6 (bottom). Although it is not as well resolved as the ambient EPR, it also clearly indicates a high-spin Co(II) center and has the same features as the ambient EPR.

Both EPR samples had the dimer species [Co_2_(μ-CO)_2_(CO)(DPPBz)_2_]^2+^ (Scheme 3) present, which was confirmed by the IR spectra run on both the samples after the EPR studies. The dimer is proposed to have a tetrahedral Co(I) center with *S* = 1, which would not be observable on the EPR instrument setup to study spin half systems.

The observation of a high-spin Co(II) catalyst was somewhat surprising because carbonyls are typically considered strong-field ligands. The localized cationic charge on the cobalt center, however, contracts the d orbitals and reduces π-backbonding to the carbonyl ligands, which is a major component of a carbonyl ligand’s strong field characteristics. The EPR likely is a mixture of the 17e^–^ [HCo(CO)_2_(DPPBz)]^+^ and 19e^–^ [HCo(CO)_3_(DPPBz)]^+^ complexes. The IR spectra (Figure 4, last spectrum) clearly show that the 2086 cm^–1^ carbonyl band assigned to the 19e^–^ [HCo(CO)_3_(DPPBz)]^+^ complex increases in intensity at lower temperatures and higher CO concentrations, conditions certainly present in the EPR studies.

The combination of the localized cationic charge and high-spin nature of the catalyst offers an excellent explanation for its high activity. The cationic charge reduces the carbonyl π-backbonding, making them more labile and allowing easier coordination of alkene and H_2_. The high-spin electronic configuration also works to weaken cobalt–ligand bonding, which appears to mainly affect the carbonyl ligands. Indeed, this is a key factor as to why chelating bisphosphine ligands are critical for this catalyst system. The chelate effect plays a key role in compensating for the high-spin electronic configuration, which also weakens the cobalt–phosphine bonding.

This is the only effective high-spin hydroformylation catalyst known, although other radical and high-spin state catalysts have been studied.^17−19^ The stability of the catalyst in the absence of excess phosphine ligand is unique among phosphine-modified cobalt and rhodium hydroformylation catalysts. This is especially odd given the high-spin nature of the catalyst that weakens the cobalt–phosphine bonding to some extent. One explanation is that dissociating the chelating bisphosphine ligand is thermodynamically very unfavorable. Neither alkene, H_2_, nor carbonyl ligands coordinate strongly enough to the cationic cobalt(II) center to compensate for the loss of the donating bisphosphine ligand under normal catalytic conditions. Co(I) and Rh(I) hydroformylation catalysts, on the other hand, are electron-rich enough to favor coordination of π-backbonding carbonyl ligands to displace phosphine ligands.

### DFT Studies and Spin State

DFT computational studies with different functionals and basis sets performed in our laboratory and at ExxonMobil used both low- and high-spin states for the [HCo(CO)_*x*_(bisphosphine)]^+^, *x* = 2–3, catalyst species.^7^ The low-spin state models generally gave structures that appeared more traditional and fit the observed carbonyl stretching frequencies better than the high-spin models (Supporting Information).

Wang and coworkers published a detailed DFT mechanistic study on the cationic Co(II) bisphosphine catalyst system using a low-spin Co(II) model.^20^ Their calculated mechanism agreed with that proposed in Figure 1: heterolytic activation of H_2_ in the key acyl to aldehyde transformation step. They calculated the transition state energies and profiles that matched the experimental data, including relative rates, quite well. They were even able to explain why more electron-donating bisphosphine ligands improve catalysis by pumping more electron-density onto the acyl ligand, which lowers the barrier for the heterolyic activation of H_2_ to eliminate aldehyde and reform the catalyst complex. This may indicate that for this class of complexes, low-spin DFT models can give good results.

### [Co(CO)_3_(DPPBz)]^+^

**Figure 7:**
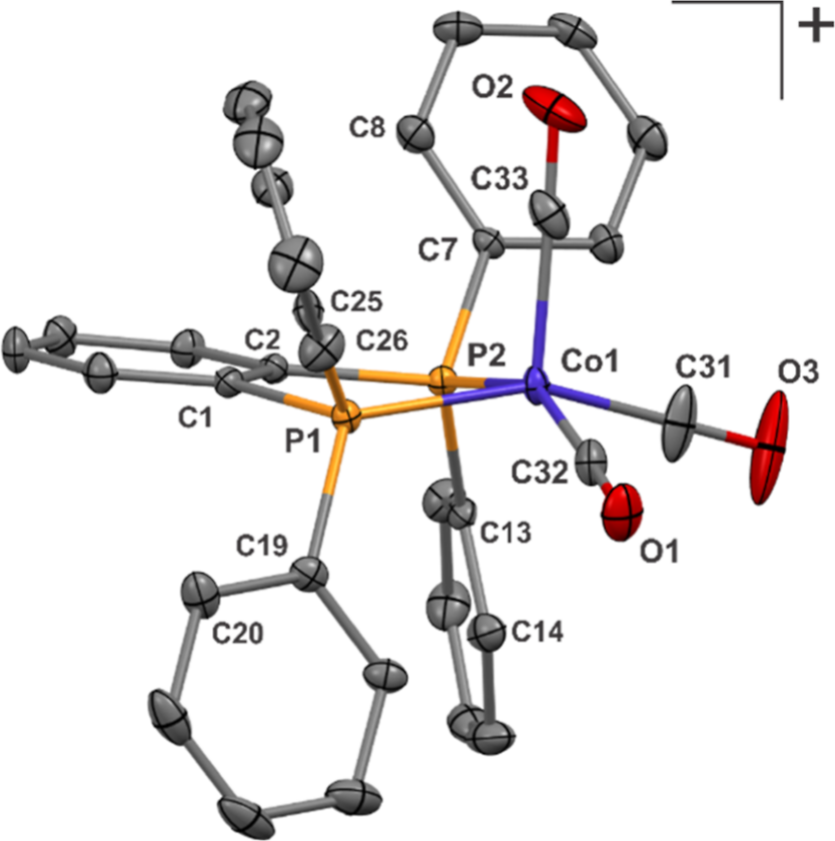
Thermal ellipsoid plot of [Co(CO)_3_(DPPBz)]^+^ grown from a high-pressure EPR tube. Hydrogen atoms, BF_4_ counter anion, and 2-methyl-THF solvent not shown for clarity. Co1–P1 = 2.198 Å, Co1–P2 = 2.207 Å, Co1–C31 = 1.79 Å, Co1–C32 = 1.78 Å, Co1–C33 = 1.84 Å.

Another high-pressure EPR quartz tube was used to activate [Co(acac)(DPPBz)](BF_4_) under H_2_/CO pressure at 120 °C in 2-methyl-THF and then placed in a freezer for several weeks. Several yellow crystals grew, which were collected after depressurization and a single-crystal structure performed (Figure 7).

The structure is a cationic Co(I) complex, [Co(CO)_3_(DPPBz)](BF_4_)·(2-methyl-THF), with a square-pyramidal geometry about the cobalt. Enough sample was collected for an IR study with carbonyl-stretching frequencies of 2094, 2050 (sh), and 2033 cm^–1^ (Supporting Information). Formation is proposed to occur from reaction of excess CO with the dimer, [Co_2_(μ-CO)_2_(CO)(DPPBz)_2_]^2+^ (Scheme 3). Previous studies with Teflon-valve sealed high-pressure tubes in our laboratory demonstrated that H_2_ seeps out more easily than CO, causing the CO/H_2_ ratio to increase over time. This increase in relative CO concentration, especially at lower temperatures, is proposed to favor cleavage of the dicationic dimer to form the cationic tricarbonyl Co(I) monomer.

The closest analog to [Co(CO_3_)(DPPBz)]^+^ is [Co(CO)_3_(^iPr^DuPhos)]^+^, (*R*,*R*)-^iPr^DuPhos = 1,2-bis((2*R*,5*R*)-2,5-diisopropylphospholano)benzene, prepared by Chirik and coworkers.^21^ This cationic Co(I) tricarbonyl bisphosphine complex also has a square pyramidal structure with quite similar bond distances and angles about the cobalt center. Lower CO-stretching frequencies of 2079, 2035, and 2011 cm^–1^ were observed due to the more strongly donating ^iPr^DuPhos ligand.

The isolation of [Co(CO_3_)(DPPBz)]^+^ supports the proposed structure for the dicationic dimer [Co_2_(μ-CO)_2_(CO)(DPPBz)_2_]^2+^. The formation of [Co(CO_3_)(DPPBz)]^+^ from the dimer occurred due to the CO-rich conditions in the EPR tube sitting for several weeks in the freezer. The highest frequency Co–CO bond seen in the in situ IR studies of the catalyst system is at 2086 cm^–1^, which has been assigned to the 19e^–^ tricarbonyl catalyst [HCo(CO)_3_(DPPBz)]^+^. The lack of a 2094-cm^–1^ carbonyl band for [Co(CO_3_)(DPPBz)]^+^ seen in the in situ FT-IR studies (Figures 2–4) indicates that the [Co_2_(μ-CO)_2_(CO)(DPPBz)_2_]^2+^ dimer does not react with CO under those conditions to form the Co(I) tricarbonyl monomer. The dimer, however, does readily react with H_2_, especially at higher temperatures, to form two [HCo(CO)_*x*_(DPPBz)]^+^ catalyst complexes.

### In Situ NMR Studies

A high-pressure 5 mm Wilmad NMR tube was used to study the catalyst system under pressure and at different temperatures. Once the catalyst precursor was loaded into the NMR tube (*d*_8_-THF solvent), it was pressurized to 10.4 bar with 1:1 H_2_/CO and then heated in an oil bath at 120 °C overnight to activate the catalyst. ^31^P NMR were collected at 24, 40, 60, 80, 100, and 120 °C—none of these spectra showed any ^31^P NMR resonances despite thousands of scans collected at each temperature. No decomposition to black cobalt metal was observed.

**Figure 8:**
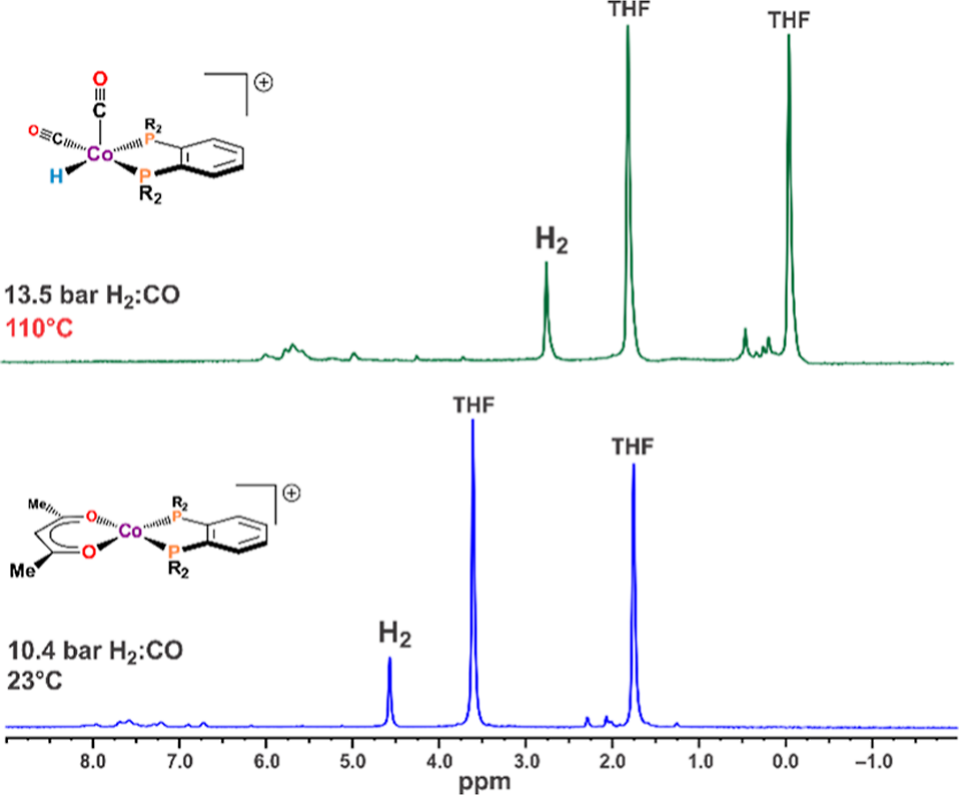
^1^H NMR in *d*_8_-THF. (Bottom) Room temperature spectrum of precursor [Co(acac)(DPPBz)]^+^ under 10.4 bar of 1:1 H_2_/CO. (Top) 110 °C spectrum after activation to form catalyst under 13.5 bar.

^1^H NMR studies were carried out at 10.4 and 24.2 bar 1:1 H_2_/CO with identical results. The results from the 10.4 bar experiment are discussed here. The ^1^H NMR was run first at room temperature to characterize the NMR of the [Co(acac)(DPPBz)](BF_4_) precursor prior to the activation. This is shown in Figure 8 (bottom). The two main peaks at 1.7 and 3.6 ppm are due to the residual proton resonances from THF, while the peak at 4.6 ppm is due to H_2_ dissolved in solution. The low intensity peaks around 2 and 7.5 ppm are due to protons on the acac and DPPBz ligands.

After activating the catalyst precursor at 110 °C for 6 h, the NMR tube was quickly transferred to the NMR probe preheated to 110 °C and the spectrum collected, as shown in Figure 8 top. The residual THF proton resonances have shifted to −0.1 and 1.8 ppm, while the H_2_ peak has shifted to 2.7 ppm. The catalyst peaks are low in intensity and shifted to around 0 and 5 ppm. The considerable shift in solvent, H_2_, and catalyst peaks is supportive of a spin-state change occurring between the precursors, [Co(acac)(DPPBz)]^+^, which is low-spin Co(II), and the catalyst, [HCo(CO)_*x*_(DPPBz)]^+^, *x* = 1–3, based on the EPR is high-spin Co(II). Some of the paramagnetic Co(I) dimer [Co_2_(μ-CO)_2_(CO)(DPPBz)_2_]^2+^, (*S* = 1), may also be present, but under these higher temperature conditions, (110 °C) the Co(II) catalysts should be the major species. The shifting of the THF and H_2_ peaks in the catalyst solution is not due to the 110 °C temperature. When the sample was cooled to room temperature, these peaks had essentially the same positions.

The NMR data supports formulation of the catalyst as a cationic high-spin Co(II) paramagnetic system [HCo(CO)_*x*_(DPPBz)]^+^, *x* = 1–3, in agreement with the EPR studies. No hydride resonance was observed at −10.7 ppm, which would indicate the formation of HCo(CO)_4_. Formation of even small amounts of HCo(CO)_4_ would release bisphosphine ligand that would be easily observable via ^31^P NMR, which was not seen.

**Figure 9:**
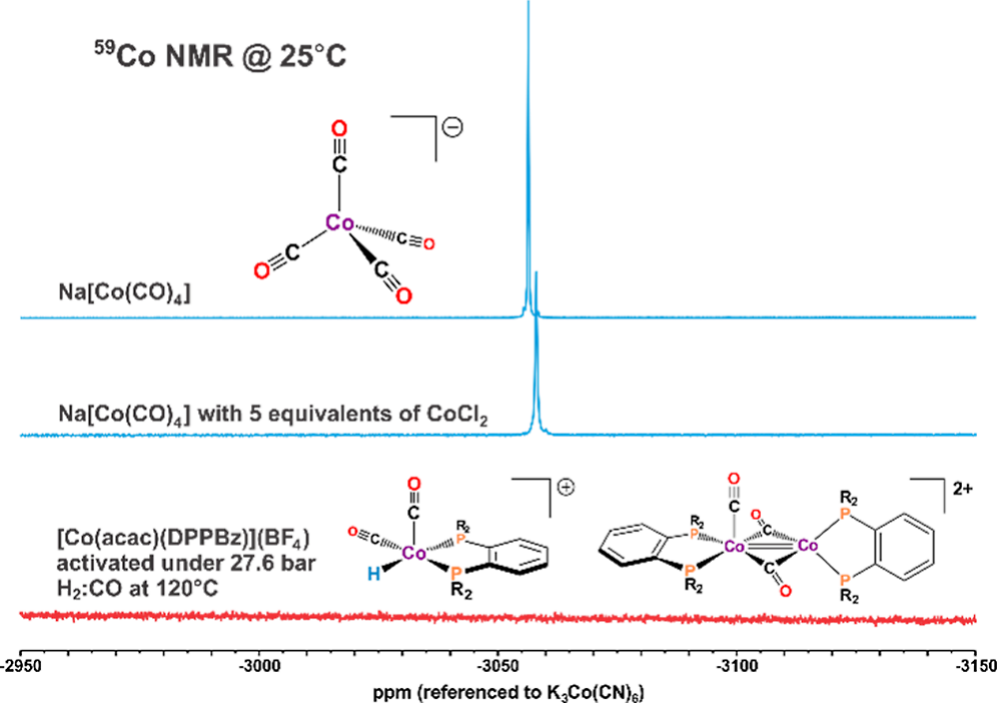
^59^Co NMR. (Top) K[Co(CO)_4_]. (Middle) K[Co(CO)_4_] with 5 equiv of CoCl_2_ added. (Bottom) Mixture of [HCo(CO)_*x*_(DPPBz)]^+^ and [Co_2_(μ-CO)_2_(CO)(DPPBz)_2_]^+^ under 27.6 bar of H_2_/CO. Reprinted in modified form with permission from ref (7).

A ^59^Co NMR study was performed to further probe if the 1888 cm^–1^ carbonyl band was due to [Co(CO)_4_]^−^, as well as if any diamagnetic Co(I) HCo(CO)_4_ or HCo(CO)_2_(bisphosphine) species were being generated. ^59^Co NMR, despite having a 7/2 quadrupolar nucleus, is quite easy to observe with a sensitivity 28% that of protons.^22−24^ K_3_[Co(CN)_6_] was used as the reference and Na[Co(CO)_4_] was prepared and studied in D_2_O. [Co(acac)(DPPBz)](BF_4_) in *d*_8_-THF was added to a high-pressure NMR tube, pressurized to 27.6 bar with H_2_/CO, and heated in an oil bath at 120 °C overnight to activate the catalyst, and then cooled to room temperature. The ^59^Co NMR spectra of [Co(CO)_4_]^−^ and the catalyst mixture at room temperature are shown in Figure 9.

Although ^59^Co is a 7/2 quadrupolar nucleus, high-symmetry cobalt complexes like [Co(CO)_4_]^−^ give a sharp singlet that is easily observed at −3056 ppm even after only five NMR pulses, as seen in Figure 9 top. Five equivalents of CoCl_2_ were added to the middle spectrum in Figure 9 to observe the effect of a paramagnetic material on observing [Co(CO)_4_]^−^. The paramagnetic cobalt(II) complex broadens and shifts the resonance somewhat for [Co(CO)_4_]^−^, but it is still easily observed after five NMR pulses. No ^59^Co resonance is observed for CoCl_2_ due to its paramagnetism.

After activating the catalyst at 120 °C overnight, it was cooled to room temperature in order to generate the 1888 cm^–1^ carbonyl species along with the [HCo(CO)_*x*_(DPPBz)]^+^, *x* = 1–3, catalyst complexes. The bottom ^59^Co NMR spectrum in Figure 9 presents 3000 scans and does not show any ^59^Co resonances over a broad range studied (5000 to −5000 ppm) for [Co(CO)_4_]^−^, HCo(CO)_4_, or HCo(CO)_2_(bisphosphine).^23,24^ After the NMR study, the IR of the catalyst mixture was taken and the 1888 cm^–1^ carbonyl band, along with the catalyst carbonyl bands between 1970 and 2086 cm^–1^ were observed.

The ^59^Co NMR data provide clear evidence that the 1888 cm^–1^ carbonyl band that forms at lower temperatures in the IR is due to the paramagnetic dicationic cobalt dimer [Co_2_(μ-CO)_2_(CO)(DPPBz)_2_]^2+^ and not [Co(CO)_4_]^−^. No diamagnetic Co(I) complexes were observed in the ^59^Co or ^31^P NMR studies, ruling out any significant participation of HCo(CO)_4_ or HCo(CO)_3_(PR_3_) type catalysts.

### Reaction Study

The question often arises as to whether the actual catalyst is present in too low concentrations to be observed spectroscopically and whether what one is observing spectroscopically are noncatalytically relevant side-equilibria. There are quite a few similarities between the cationic Co(II) bisphosphine catalyst [HCo(CO)_*x*_(DPPBz)]^+^, *x* = 1–3, and HCo(CO)_4_. Both are highly active toward sterically hindered branched internal alkenes that are very difficult to hydroformylate.^7^ They both have similarly low aldehyde regioselectivities for 1-alkenes such as 1-hexene (∼1:1 L/B for the bisphosphine ligands studied) and are highly active alkene isomerization catalysts.

The main difference is the higher stability of [HCo(CO)_*x*_(bisphosphine)]^+^ toward decomposition to cobalt metal under a variety of temperature–pressure conditions and the greater ability to hydrogenate aldehyde to alcohol when the aldehyde concentration is high relative to alkene. The cationic Co(II) bisphosphine catalyst system has a maximum operating temperature of around 170–180 °C at the highest pressure studied (90 bar).

The possibility of HCo(CO)_4_ being formed from [HCo(CO)_*x*_(DPPBz)]^+^ was probed by adding monodentate phosphine. The Shell phosphine-modified cobalt catalyst is essentially generated by adding an alkylated sterically bulky phosphine ligand to HCo(CO)_4_ to form HCo(CO)_3_(PR_3_). This generates a much slower hydroformylation catalyst, but one that produces L/B aldehyde selectivities around 6–8:1 compared to only around 1:1 for HCo(CO)_4_.^4^ The phosphine ligand also makes it a better hydrogenation catalyst, which converts aldehyde to alcohol (desired), as well as alkene to alkane (undesired).

A 1-hexene hydroformylation run was performed using the phosphine-modified cobalt(I) catalyst system: Co(acac)_2_ and 3 equiv of PBu_3_ as the model phosphine (2 mM Co, 180 °C, 50 bar H_2_/CO, 6 mM PBu_3_, 1.0 M 1-hexene, dimethoxytetraglyme). The temperature was increased to 180 °C and the catalyst concentration was doubled due to the slowness of the phosphine-modified cobalt catalyst. Only a 1:1 H_2_/CO ratio was used to limit the aldehyde and alkene hydrogenation reactions. The initial TOF was 4.5/min and after an hour, the following was observed: 6:1 L/B aldehyde regioselectivity, 24.6% aldehyde, 60.3% alkene isomerization, 4% alcohol, and 1% alkane.

1 equiv of PBu_3_ was then used to probe its effect on the [HCo(CO)_*x*_(DPPBz)]^+^ catalyst system (Table 1). The only effect of the added PBu_3_ was to slow the cationic [HCo(CO)_*x*_(DPPBz)]^+^ catalyst down by about half and reduce alkene isomerization. Both are expected if the PBu_3_ is only acting as an inhibitory ligand blocking the equatorial site on the cationic Co(II) catalyst center from alkene coordination. Similar inhibitory results were obtained using 10% acetonitrile by solvent volume as shown in Table 1.

**Table 1:** 1-Hexene Reaction with [HCo(CO)_*x*_(DPPBz)]^+^^a^

additive	TOF (min^–1^)	L/B	% aldehyde	% iso	% hydro
	45.4	1.1	45.4	38.1	0.8
PBu_3_	23.8	1.1	23.8	26.0	0.5
N≡CCH_3_	23.1	0.8	23.1	19.7	0.4

a 1 mM [Co(acac)(DPPBz)](BF_4_), 1.0 M 1-hexene, 160 °C, 50 bar 1:1 H_2_/CO, dimethyoxytetraglyme solvent. 1 equiv of PBu_3_ used. 10% acetonitrile by solvent volume used. Results after 10 min. TOF = initial turnover frequency, L/B = linear to branched aldehyde ratio; iso = alkene isomerization, hydro = alkene hydrogenation. No aldehyde hydrogenation to alcohol observed.

Adding PBu_3_ to the cationic Co(II) bisphosphine catalyst system (Table 1) did not generate the Shell phosphine-modified Co(I) catalyst, which would have been reflected in far slower reaction rates and higher aldehyde L/B regioselectivity with 1-hexene as the substrate. This indicates that [HCo(CO)_*x*_(DPPBz)]^+^ was not falling apart to make HCo(CO)_4_ as that would have reacted with the PBu_3_ to generate HCo(CO)_3_(PBu_3_).

The original studies demonstrated that chelating phosphines were critically important and that monodentate phosphines did not work with the cationic Co(II) catalyst system.^7^ Furthermore, the strongly donating alkylated-chelating phosphines such as depe and (Et_2_P)_2_-1,2-C_6_H_4_ (DEPBz) generated the most active cationic Co(II) hydroformylation catalysts. This is exceptionally unusual as there are no examples of either Rh(I) or Co(I) hydroformylation catalysts where electron-donating phosphines generate more active hydroformylation catalysts. Indeed, exactly the opposite is well known, electron-donating chelating phosphines strongly inhibit Co(I) or Rh(I) for hydroformylation.^25^

Chirik and coworkers, for example, recently reported a neutral Co(I) hydroformylation catalyst that can also hydrogenate aldehydes to alcohols based on the electron-donating dicyclohexylphosphinoethane (dcype) ligand, HCo(CO)_2_(dcype).^26^ However, this catalyst is inactive under normal thermal hydroformylation conditions and requires photolysis to work. Impressively, this system can get over 99:1 L/B regioselectivity for producing alcohol, but only does 20 turnovers over 48 h using blue LED (427 nm) at 42 °C using 4 bar of 9:1 CO/H_2_. The high CO ratio was utilized to minimize alkene hydrogenation. This is an example of how electron-donating bisphosphines increase Co–CO π-backbonding and strongly inhibit thermal dissociative reactions.

### Catalyst Degradation

The [HCo(CO)_*x*_(bisphosphine)]^+^, *x* = 1–3, catalyst system has remarkable stability as indicated by (1) the lack of cobalt-induced phosphine ligand fragmentation reactions observed so far; (2) no excess bisphosphine ligand used in the catalysis runs; (3) high turnover numbers possible (over 1 million); and (4) no sign of catalyst degradation under operating conditions without alkene present over a 100-h period (Figures 2–4).

Rh(I) and Co(I) phosphine-based hydroformylation catalysts require some excess of phosphine ligand present in order to maintain moderate catalyst stability and regioselectivity. This is because phosphines that generate active and/or selective rhodium catalysts are poor-to-moderate σ-donors and have facile dissociation equilibria under catalytic conditions. Even Rh(I) hydroformylation catalysts that use chelating bisphosphines are typically run with 3–5 equiv of excess bisphosphine ligand.^27^

The high activity of Rh(I) often leads to rhodium-induced phosphine ligand and catalyst degradation reactions, especially for phosphines with phenyl, benzyl, or alkoxide groups.^11,12^ Rh-phosphine degradation reactions are especially serious when the catalyst is under operating conditions without any alkene substrates present. This is one reason why the stability shown by the cationic Co(II) bisphosphine catalyst in over a 100-h in situ FT-IR study is so impressive.

The cationic Co(II) bisphosphine catalyst system can degrade, however, especially at higher temperatures (e.g., >160 °C), if there is not enough H_2_ and CO present. A small amount of decomposition to cobalt black, for example, was seen for the hydroformylation of 1-hexene using the DPPBz-based cationic Co(II) catalyst at 160 °C and 30 bar of H_2_/CO.^7^

**Figure 10:**
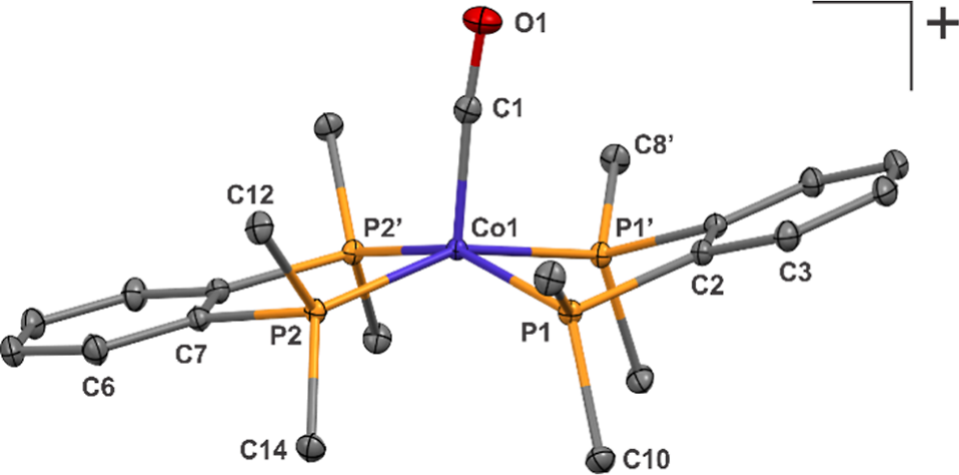
Thermal ellipsoid plot of [Co(CO)(DEPBz)_2_]^+^. Hydrogen atoms, methyl carbons, and BF_4_ counteranion omitted for clarity. There is a mirror plane bisecting the phosphine ligands and containing the cobalt carbonyl. Co1–P1 = 2.208 Å, Co1–P2 = 2.203 Å, Co1–C1 = 1.79 Å.

The primary molecular decomposition species identified so far is the cationic Co(I) complex with two chelating bisphosphine ligands: [Co(CO)(bisphosphine)_2_](BF_4_) (bisphosphine = DPPBz, DEPBz, and dppe). The structure of the red-orange [Co(CO)(DEPBz)_2_]^+^ complex is shown in Figure 10. One X-ray quality crystal of the double DEPBz complex was isolated from an in situ ReactIR study where 1:1 H_2_/CO followed by pure CO atmospheres were used. Not enough of this complex was isolated for separate IR or NMR characterization. The complex has very low solubility in the dimethoxytetraglyme solvent used for the ReactIR study.

The [Co(CO)(DPPBz)_2_]^+^ complex was initially observed from an in situ ReactIR study of the DPPBz-based cationic cobalt(II) catalyst system. After each ReactIR study, the high-pressure ATR cell is taken apart and cleaned. Small quantities of red-orange microcrystals of [Co(CO)(DPPBz)_2_]^+^ were formed on the Teflon ring seal at the base of the silicon ATR probe that inserts into the bottom of the high-pressure IR cell. There is a very small gap between the silicon ATR cylindrical probe and the inner wall of the IR cell, which allows a small amount of reaction solution to seep down to the Teflon seal. The temperature at the Teflon seal is higher than the main cell solution and there is extremely poor gas transport or mixing in this region. The higher temperature and low gas concentration at the Teflon seal is apparently conducive for forming these double-bisphosphine cobalt complexes. The IR spectrum (Supporting Information) of [Co(CO)(DPPBz)_2_]^+^ was measured directly from the red-orange microcrystalline deposits on the Teflon seal with ν_CO_ = 1910 cm^–1^.

The other case where a considerable amount of [Co(CO)(DPPBz)_2_]^+^ was produced occurred after activating the DPPBz-based catalyst precursor with H_2_/CO in the autoclave and was then allowed to sit overnight under pure carbon monoxide before purging and refilling with H_2_/CO. The hydroformylation run attempted with this did not work and orange-red crystalline material was found after opening the autoclave. The IR on this material also showed a strong CO band at 1910 cm^–1^ demonstrating that it was the cationic Co(I) [Co(CO)(DPPBz)_2_]^+^ complex. The ^31^P NMR in *d*_3_-acetonitrile showed a sharp singlet at 62 ppm, consistent with the structures seen for the DEPBz and dppe double-ligand complexes.

The structure of [Co(CO)(DPPBz)_2_](PF_6_) was reported by Chow and coworkers.^28^ Interestingly, it has a trigonal pyramidal coordination geometry, with the CO ligand in the equatorial site, unlike the square-pyramidal geometry seen for the [Co(CO)(DEPBz)_2_](BF_4_) structure (Figure 10). They report a ν_CO_ = 1927 cm^–1^ unlike the 1910 cm^–1^ value observed for both of our red-orange [Co(CO)(DPPBz)_2_](BF_4_) products formed in dimethoxytetraglyme solvent. The double-DPPBz ligated complexes with the 1910 cm^–1^ carbonyl-stretching frequency is proposed to have the same square-pyramidal structure seen for the DEPBz complex (Figure 10), which explains the difference in CO-stretching frequencies.

[Co(CO)(DPPBz)_2_]^+^ has not been observed in any of the in situ IR studies using the DPPBz catalyst precursor, either by the distinctive ν_CO_ band at 1910 cm^–1^, or by formation of the mostly insoluble red-orange crystals (or solid) in the main reaction solution. Nor has the distinctive red-orange microcrystalline solid been observed in any of the hundreds of successful hydroformylation runs performed in our laboratory.

The double-bisphosphine-chelated DPPBz complex [Co(CO)(DPPBz)_2_](BF_4_) is inactive for hydroformylation and offers one explanation for why adding excess amounts of bisphosphine ligand slows or deactivates the cationic Co(II) catalyst system.

### Catalyst Precursor Problem

**Scheme 4:**
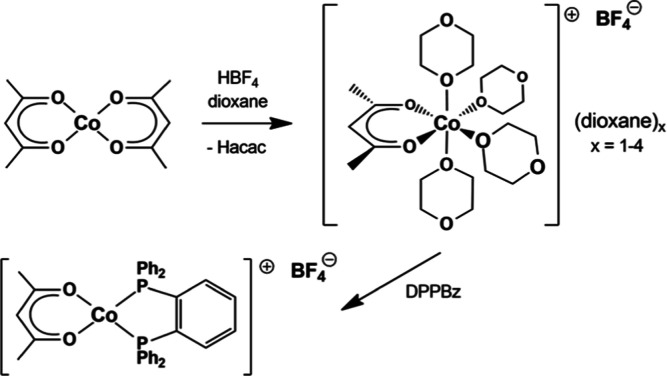
Catalyst precursor synthesis.

The synthesis of the catalyst precursor [Co(acac)(DPPBz)](BF_4_) is shown in Scheme 4. Franke and Zhang reported that the [Co(acac)(DPPBz)](BF_4_) catalyst precursor prepared in their laboratory did not generate an active hydroformylation catalyst.^8^ A comparison of the spectroscopic data reported by Franke and Zhang for the DPPBz-based catalyst precursor does not match what we reported.^7^ The details are presented in the Supporting Information. This clearly indicates, therefore, that they did not synthesize the [Co(acac)(DPPBz)](BF_4_) catalyst precursor.

The starting material that we used for preparing the cationic Co(II) precursor was [Co(acac)(dioxane)_4_](BF_4_)·(dioxane)_*x*_, *x* = 1–4. Franke and Zhang reported that they used an oligomeric cationic Co(II)–acac dioxane complex with bridging dioxanes between the cobalt centers and approximately 31 dioxanes per cobalt.^8^ The large excess of dioxanes proposed to be present argues strongly against an oligomeric structure and calls into question the nature of what they actually used as the cationic Co(II) starting material.

The synthetic procedure we published for preparing [Co(acac)(dioxane)_4_](BF_4_)·(dioxane)_*x*_ stated that the quality and concentration of the HBF_4_·etherate used was critically important (Supporting Information).^7^ This may well be the core problem with the synthetic issues that Franke and Zhang encountered in preparing the cationic Co(II) bisphosphine catalyst precursor.

## Conclusion

The spectroscopic and reaction studies presented fully support the proposed cationic Co(II) bisphosphine catalyst system: [HCo(CO)_*x*_(bisphosphine)](BF_4_), *x* = 1–3. The EPR studies indicate a high-spin, *S* = 3/2, catalyst. The spectroscopic and reaction studies do not support the formation or the role of Co(I) catalysts such as HCo(CO)_4_, HCo(CO)_3_(phosphine) or the Co(−1) complex [Co(CO)_4_]^−^. The combination of a localized cationic charge on the cobalt center along with the high-spin electronic configuration offers an excellent explanation for the high activity of this catalyst system. The localized cationic charge on the cobalt contracts the d-orbitals and reduces the carbonyl π-backbonding, which weakens the Co–carbonyl bonding and facilitates CO dissociation. The same role of localized cationic charge increasing the hydroformylation activity has been observed for the dirhodium tetraphosphine catalyst system studied by our group.^29,30^

The high-spin electronic configuration adds to the weakening of the Co–carbonyl bonding and contributes to the high activity. The chelating bisphosphine ligand plays a critical role via stronger coordination to compensate for the high-spin weakening of the Co–phosphine bonding. Monodentate phosphine ligands do not generate active cationic Co(II) hydroformylation catalysts.^7^ Electron-donating bisphosphine ligands generate more active cationic Co(II) catalysts by increasing the electron-density on the hydride and transferring more electron density to the acyl ligand. Increasing the partial negative charge on the acyl ligand lowers the activation barrier for the heterolytic cleavage of H_2_ and elimination of the aldehyde product.^20^

Electron-donating bisphosphine ligands also increase the partial negative charge on the catalyst precursor’s acac ligand, [Co(acac)(bisphosphine)]^+^, allowing easier and lower temperature heterolytic activation of H_2_ to generate the catalyst. For example, the DPPBz-based Co(II) catalyst precursor requires temperatures of 120–140 °C to quickly activate, while the stronger donating DEPBz or depe catalyst precursors activate rapidly around 100 °C.

The ability to form 19e^–^ complexes plays an important role in weakening the stronger coordinated equatorial Co–CO ligand to enhance dissociation and allow coordination of alkene to the sterically preferred equatorial site. Basolo demonstrated that the carbonyl substitution chemistry for the 17e^–^ V(CO)_6_ radical proceeds 10^10^ times faster than for 18e^–^ Cr(CO)_6_.^31^ The phosphine substitution reaction with the 17e^–^ V(CO)_6_ radical proceeding through a 19e^–^ transition state was shown to be associative and extremely facile. The 18e^–^ [V(CO)_6_]^−^ anion, in marked contrast, is inert toward phosphine substitution reactions.

Although the [HCo(CO)_*x*_(bisphosphine)]^+^, *x* = 1–3, catalyst system uses many of the same mechanistic steps seen for other hydroformylation catalysts, there are several unique aspects. The first being that H_2_ is activated via a heterolytic cleavage mechanism, which does not involve any change in the metal center’s oxidation state. The radical nature of the catalyst allows the formation of 19e^–^ complexes that play an important role in weakening the Co–carbonyl bonding and enhancing carbonyl dissociation, especially from the key equatorial Co–CO coordination site. The localized cationic charge and high-spin electronic state of the cobalt center also work together to weaken the Co–carbonyl bonding, increasing the activity of the catalyst.

The heterolytic H_2_ activation combined with the various electronic factors discussed above enables higher catalytic activity with electron-rich chelating bisphosphine ligands. This is unique for any other known hydroformylation catalyst system, especially since high catalyst stability is seen with no excess phosphine ligand. Although the cationic Co(II) catalyst system currently has low L/B aldehyde selectivity with 1-hexene for the limited number of chelating bisphosphine ligands studied, there is certainly potential for developing catalysts with much higher L/B selectivity.

## Supporting Information Available

General information and details on spectroscopic, catalytic runs, reaction studies, crystallographic details, and DFT calculations (PDF)
