# Supplementary material for: Cationic Cobalt(II) Bisphosphine Hydroformylation Catalysis: In Situ Spectroscopic and Reaction Studies
Source: J Am Chem Soc. 2023 Aug 29;145(36):19715–26. doi: 10.1021/jacs.3c04866 (PMC11864568; doi:10.1021/jacs.3c04866)
Supplement: Supplementary file 1 — ja3c04866_si_001.pdf [file ja3c04866_si_001.pdf]

Cationic cobalt(II) bisphosphine hydroformylation catalysis: *in situ* spectroscopic and reaction studies

Drew M. Hood, Ryan A. Johnson, David J. Vinyard,† Frank R. Fronczek, George G. Stanley*

Department of Chemistry, Louisiana State University, Baton Rouge, Louisiana, 70803-1804, United States

†Department of Biological Sciences, Louisiana State University, Baton Rouge, Louisiana, 70803-1804, United States

Correspondence to: gstanley@lsu.edu

General Information 3

$(Et_2P)_2$-1,2-C_6H_4, DEPBz Bisphosphine Ligand Synthesis 4

[Co(acac)(dioxane)$_4$](BF_4)•(dioxane)$_x$ ($x \approx$ 1-4) 4

[Co(acac)(bisphosphine)](BF_4) General Procedure 5

[Co(acac)(DPPBz)]BF_4 6

[Co(acac)(dppe)]BF_4 7

[Co(acac)(depe)]BF_4 7

[Co(acac)(DEPBz)]BF_4 7

Fig. S1. FT-IR spectra of [Co(acac)(DPPBz)](BF_4), [Co(acac)(dppe)](BF_4), and [Co(acac)(depe)](BF_4) 9

Fig. S2. FT-IR spectra of [Co(acac)(DEPBz)](BF_4) and starting materials 10

Fig. S3. FT-IR spectrum of [Co(CO)$_3$(DPPBz)](BF_4). 11

Fig. S4. FT-IR spectrum of [Co(CO)(DPPBz)$_2$](BF_4). 12

Fig. S5. ^{1}H NMR of [Co(acac)(DPPBz)](BF_4) in acetone-d_6 13

Fig. S6. ^{13}C NMR of [Co(acac)(DPPBz)](BF_4) in $CDCl_3$ 14

Fig. S7. ^{1}H NMR spectrum of [Co(acac)(depe)](BF_4) in acetone-d_6 15

Fig. S8. ^{1}H NMR spectrum of [Co(acac)(dppe)](BF_4) in acetone-d_6 16

Fig. S9. High resolution electrospray mass spectrum of [Co(acac)(DPPBz)]$^+$ 17

Fig. S10. High resolution electrospray mass spectrum of [Co(acac)(dppe)]$^+$ 18

LSU Hydroformylation Experiments 19

Fig. S11. Schematic drawing of one of the Parr autoclave systems 21

Fig. S12. Photograph of two of the Parr autoclave systems 22

Fig. S13. ReactIR 101 hour study of [Co(acac)(DPPBz)](BF_4): 2.4 to 6.8 hrs 23

Fig. S14. ReactIR 101 hour study of [Co(acac)(DPPBz)](BF_4): 6.8 to 28.9 hrs 24
Fig. S15. ReactIR 101 hour study of [Co(acac)(DPPBz)](BF_4): 28.9 to 33.0 hrs 25
Fig. S16. ReactIR 101 hour study of [Co(acac)(DPPBz)](BF_4): 53.1 to 96.4 hrs 26
Fig. S17. ReactIR 101 hour study of [Co(acac)(DPPBz)](BF_4): 96.1 to 101.5 hrs 27
Crystallographic Structure Determination 28
Table S1. Crystal Data and Structure Refinement 28
Fig. S18. Thermal ellipsoid plot of [Co(CO)($dppe_2$)](BF_4)•(toluene). 29
Fig. S19. FT-IR comparison of [Co(acac)(DPPBz)](BF_4) catalyst precursors. 30
Fig. S20. ^{1}H NMR comparison of [Co(acac)(DPPBz)](BF_4) catalyst precursors. 31
Fig. S21. Proposed structure of [$Co_m(acac)_m(\mu$-dioxane$)_n$]$(BF_4)_m$
and ^{13}C NMR by Franke & Zhang. 32
Fig. S22. Gaussian 16 DFT calculated structures for [$HCo(CO)_2$(DMPBz)]$^+$
using low-spin and high-spin models. 33
Fig. S23. Gaussian 16 DFT calculated structure for [$Co_2(\mu$-CO$)_2$(CO)$(DMPBz)_2$]$^{2+}$ 34

General Information

All manipulations of air- and moisture-sensitive reagents were performed under an inert atmosphere of nitrogen in either a Vacuum Atmospheres or MBraun Glovebox or using standard Schlenk techniques. All solvents were reagent grade or higher. When dealing with air-sensitive reagents, the solvents were degassed with nitrogen prior to use. Chemicals used were the highest purity available from Aldrich or Strem Chemicals and used as received (degassed with nitrogen as needed).

^{31}P, ^{59}Co, and ^{1}H NMR spectra were recorded on either a Bruker AV-400 or AVIII-400 spectrometer. All ^{1}H NMR spectra were referenced internally to either added TMS (0.0 ppm) or to the residual solvent peak. All ^{31}P NMR spectra were referenced externally to 85% H_3PO_4 (0.0 ppm). ^{59}Co NMR were referenced to $K_3[Co(CN)_6]$ (0.0 ppm). NMR data processing was done using Bruker Topspin v3.4 or MestReNova v11.0 software packages. Hydroformylation samples were analyzed on an Agilent 5975B/6890N GC/MS using a HP-5MS 30 m 0.25 micron column. High resolution mass spectra were collected on an Agilent 6230 Electrospray TOF instrument in positive ion mode via direct injection of the sample dissolved in a 60:40 solvent system of acetonitrile and 0.1% formic acid/water. Elemental analysis performed by Midwest Microlab, Indianapolis, IN.

FT-IR spectra collected on a Bruker Tensor 27 instrument equipped with a TDGS room-temperature detector. Bruker OPUS v8.0 software was used for data collection, processing, and curve-fitting. High-pressure and variable temperature FT-IR spectra collected on a Mettler-Toledo ReactIR model 45m equipped with a liquid-nitrogen cooled MCT detector. This was connected with an AgX fiber optic conduit to a Mettler-Toledo/Parr high pressure IR cell that used a SiComp (silicon ATR) Sentinel probe. The high-pressure IR cell was modified with a Teflon gasket for the SiComp probe seal to the main cell body. This all-Teflon gasket makes a much better pressure seal compared to the original gasket that came with the IR cell. The head-piece of the IR cell was modified with Swagelok quick-connects equipped with solvent-resistant Markez O-rings to facilitate assembly and cleaning. Mettler-Toledo iC IR v7.0 software was used for data collection. Bruker OPUS v8.0 software was used to do baseline corrections on the data collected from the ReactIR system as well as curve-fitting the carbonyl region.

EPR spectra were recorded on a Bruker EMX spectrometer equipped with a standard ER-4102 resonator and an Oxford ESR-900 helium flow cryostat. Acquisition parameters were frequency, 9.475 GHz; modulation amplitude, 10 G; modulation frequency, 100 kHz; time constant, 164 ms; conversion time, 41 ms; sweep time, 84 s. EPR spectra were simulated using the “pepper” function in EasySpin version 5.2.20.

Single-crystal X-ray structures were collected on at low temperature on a Bruker Kappa Apex-II DUO CCD diffractometer fitted with an Oxford Cryostream chiller. Radiation was MoKα, from a fine-focus sealed tube with Triumph curved graphite monochromator.

Catalytic reactions were done in 160 mL Parr autoclaves modified with Swagelok quick-connects with solvent-resistant Markez O-rings for gas and other related connections to the autoclave, which allowed easy disassembly and cleaning. A Parr 4870 controller is interfaced with four autoclave systems and a Windows PC computer running SpecView v2.5 software to collect and analyze the temperatures, pressures, stirring rates, and times of catalytic runs. A schematic diagram of one autoclave system is shown in Figure S9 and a photograph of two autoclaves that share the three gas reservoirs in one hood is shown in Figure S10.

$(Et_2P)_2$-1,2-C_6H_4, DEPBz Bisphosphine Ligand Synthesis:

1-(Et_2P)-2-iodobenzene precursor:

The following procedure was conducted in aluminum foil-wrapped glassware to exclude light. A Schlenk flask was charged with 25.00 g (75.8 mmoles) of 1,2-diiodobenzene and 80 mL of THF and cooled in an ice bath. A second Schlenk flask was charged with 26.1 mL (75.8 mmoles) of a 2.9 M THF solution of *i*PrMgBr. The room temperature Grignard solution was added dropwise to the 0°C flask with the 1,2-diiodobenzene via cannula. It is important that the Grignard flask is kept at room temperature to ensure that the Grignard does not precipitate out of solution. The cooled reaction flask was stirred at 0ºC for ~18 hours (overnight) using a large wide-mouth dewar filled with ice. The reaction flask is then cooled to −25°C and a solution of Et_2PCl (9.72 g, 78 mmoles) dissolved in 90 mL of THF was added dropwise via cannula. The yellow solution was then allowed to warm to room temperature and stirred overnight. The next day 80 mL of O_2-degassed water was added and the organic layer was separated. The aqueous layer was extracted with three 50 mL portions of diethyl ether. The extracts and organic layer were combined and dried over Na_2SO_4. Solvents were removed under vacuum leaving a yellow oil. The product was isolated via short-path distillation to yield 16.0 g of an air- and light-sensitive colorless liquid (72% yield). Yields are usually between 70-75% and purity is usually greater than 99% via ^{31}P NMR. ^{1}H NMR (400.130 MHz, C_6D_6): δ = 7.7 (br m, 1H), 7.2 (sharp m, J = 7.3 Hz, 2H), 6.8 (sharp m, J = 7.3 Hz, 1H), 1.5 (m, 4H), and 0.9 ppm (m, J = 7.3 Hz, $J_{P,H}$ = 7.7 Hz, 6H). $^{13}C\{^{1}H\}$ NMR (62.895 MHz, C_6D_6): δ = 142.3 (d, J_{P-H} = 15.3 Hz), 139.5 (s), 139.4 (d, J_{P-H} = 15.3 Hz), 108.5 (d, J_{P-H} = 40.3 Hz), 77.4 (d, J_{P-H} = 30.7 Hz), 76.6 (s), 19.3, and 9.5 ppm (d, J_{P-H} = 13.4 Hz). $^{31}P\{^{1}H\}$ NMR (161.976 MHz, C_6D_6): δ = 0.3 ppm (s).

DEPBz Ligand Synthesis:

A Schlenk flask was charged with 8.0 g (27.4 mmoles) of 1-(Et_2P)-2-iodobenzene and 20 mL of THF and cooled in an ice bath. A second Schlenk flask was charged with 9.17 mL (69.3 mmoles) of a 2.9 M THF solution of *i*PrMgBr. The room temperature Grignard solution was added dropwise to the 0°C flask with the 1-(diethylphosphino)-2-iodobenzene via cannula. The cooled reaction flask was stirred at 0ºC for ~18 hours (overnight). The reaction flask is then cooled to −25°C and a solution of Et_2PCl (3.4 g, 27.4 mmoles) dissolved in 30 mL of THF was added dropwise via cannula. The solution was then allowed to warm to room temperature and stirred overnight. The next day 40 mL of O_2-degassed water was added and the organic layer was separated. The aqueous layer was extracted with three 50 mL portions of diethyl ether. The extracts and organic layer were combined and dried over Na_2SO_4. Solvents were removed under vacuum. Yields are usually between 73-78%. ^{1}H NMR (400 MHz, C_6D_6) δ = 7.29 (dt, J_{P-H} = 6.1, 3.1 Hz, 2H), 7.15 (d, J_{P-H} = 5.6 Hz, 2H), 1.82 – 1.52 (m, 8H), 1.56 – 1.35 (m, 2H), 0.94 (ddt, J_{P-H} = 32.5, 15.1, 7.7 Hz, 12H). $^{31}P\{^{1}H\}$ NMR (161.976 MHz, acetone-d_6): δ = –27.6 ppm.

[Co(acac)(dioxane)$_4$](BF_4)•(dioxane)$_x$ (x ≈ 1-4):

5 g (19.4 mmoles) of Co(acac)$_2$ (acac = acetoacetonate) along with 150 mL of dioxane is added to a 500 mL two neck Schlenk flask equipped with a condenser. The solution is heated to 60°C with stirring until the Co(acac)$_2$ has dissolved. Then the solution is cooled to 50-45°C

before 3.3 g (20.4 mmoles, 1.05 equivalents) of HBF_4•Et_2O is added dropwise to the solution via cannula.

> ***Important Note:*** the tetrafluoroboric acid ether complex has to be relatively fresh. We have observed that if the acid is more than a month or two old the acid sometimes does not yield clean enough product. We recently received HBF_4•Et_2O from a supplier that was a light tan-brown color. This HBF_4•Et_2O did not work as well in our synthesis relative to the previous darker tan-brown HBF_4•Et_2O material received.

The resulting solution is allowed to stir overnight while returning to room temperature. The pink precipitate is collected on a Schlenk glass frit and washed with diethyl ether. The resulting pink powder is then dried under vacuum overnight to remove excess dioxane to yield 6.5 g of material (7.6 mmol, 39%). Typical isolated yields = 35-45%. The lower isolated yield is mainly due to inefficiency in removing the solid product from the Schlenk frit in a glove box. ^{1}H NMR (400 MHz, D_2O, 24°C): δ = 4.74 ($\Delta v_{1/2}$ = 14 Hz), 3.64 ($\Delta v_{1/2}$ = 5.1 Hz), 2.18 ($\Delta v_{1/2}$ = 5.1 Hz). ^{13}C{^{1}H} NMR (100.613 MHz, D_2O, 24°C): δ = 208.6 ($\Delta v_{1/2}$ = 5.7 Hz), 66.2 ($\Delta v_{1/2}$ = 2.8 Hz), 30.4 ($\Delta v_{1/2}$ = 3.7 Hz). IR (cm^{-1}, diamond ATR cell, water present due to running sample in air, see Figure S2): 3499.5 (br, m), 3233.7 (sh, br, w), 2973.1 (w), 2927.4 (w), 2875.3 (w), 1721.7 (w), 1652.2 (w), 1582.0 (m), 1522.9 (vs), 1456.4 (m), 1358.1 (s), 1258.9 (m), 1096.7 (vs), 1072.3 (vs), 1044.2 (sh, w), 1017.2 (vs), 938.2 (s), 896.6 (m), 862.3 (vs), 784.3 (w), 755.8 (m).

In order to obtain a final molecular weight for synthesis applications a ^{1}H NMR should be performed using D_2O as a solvent in order to determine the amount of dioxane solvent that is present. The dioxane and acetylacetonate peaks are integrated and their respected areas correlated as shown below. The molecular weight of the starting material complex is calculated and used for further synthesis. The dioxane and acetylacetonate ^{1}H NMR resonances are integrated and their relative integrated peak areas correlated as shown below. The molecular weight of the [Co(acac)(dioxane)$_4$](BF_4)•(dioxane)$_x$ (x ≈ 1-4) complex is then calculated and used for further syntheses.

$$\text{\# of Dioxanes} = \frac{6(\text{area of Dioxane NMR peaks})}{8(\text{area of Acetylacetonate NMR peaks})}$$

Note that the solvated dioxane is slowly lost as this compound is manipulated in a glove box, so the calculated molecular weight does not stay constant over longer periods of time and exposure to the glove box atmosphere. The molecular weight should be rechecked by ^{1}H NMR spectroscopy if more than a week or so of glove box use has passed since the last calculation. Note that high dioxane to Co ratios (>10) indicate poor starting material and reduced or poor catalyst performance was observed with attempts to make catalyst precursor with such material.

[Co(acac)(bisphosphine)](BF_4) General Procedure:

All manipulations of air- and moisture-sensitive reagents were performed under an inert atmosphere of nitrogen in either a Vacuum Atmospheres or MBraun Glovebox or using standard Schlenk techniques. The catalyst precursor of a desired bisphosphine ligand is made by adding one equivalent of ligand dissolved in CH_2Cl_2 to the [Co(acac)(dioxane)$_4$](BF_4)•(dioxane)$_x$ (x ≈ 1-

4) complex dissolved in acetone (0.1 g/20-30 mL acetone). The resulting solution is stirred for 30 mins and the solvent is then removed under vacuum. If the resulting solid is tacky (this occurs when considerable amounts of dioxane are present in the starting [Co(acac)(dioxane)$_4$](BF_4)•(dioxane)$_x$ (x ≈ 1-4) complex) then dissolve the material in CH_2Cl_2 before removing the solvent under vacuum again. The resulting powder should be red to brown depending on the bisphosphine ligand that is coordinated. Reaction yields are typically 90+%, although isolated yields are always lower due to the difficulty in removing all the powdered product from the reaction flask in the glove box. Based on the ^{1}H NMR, some dioxane solvent can be carried over into the isolated [Co(acac)(bisphosphine)](BF_4) complexes. The ^{1}H NMR dioxane peak intensities observed indicate that the maximum amount of dioxane present is approximately one per [Co(acac)(bisphosphine)](BF_4) complex, but is often less. We, therefore, do not include dioxane in our MW catalyst precursor calculations for catalytic runs.

Due to the paramagnetism of the Co(II) complex some ^{1}H NMR data can be collected, but ligand resonances are considerably broadened and paramagnetically shifted. No ^{31}P NMR data could be collected due to the paramagnetism of the catalyst precursors that completely wiped out the ^{31}P resonances. FT-IR spectra of the catalyst precursors are shown in Figures S1 and S2. High resolution mass spectral data on [Co(acac)(DPPBz)](BF_4) and [Co(acac)(dppe)](BF_4) catalyst precursor species were collected and supports their formulation (Figures S7 and S8). The EPR spectrum of [Co(acac)(DPPBz)](BF_4) shows a pure complex with respect to other Co(II) species that could be present. Obtaining crystalline samples of the precursors has proved extremely difficult so far, only a few crystals of the [Co(acac)(DPPBz)](BF_4)•THF complex have been obtained. All catalytic runs used powdered samples directly from the various cobalt(II) precursor syntheses.

[Co(acac)(DPPBz)]BF_4:

0.100 g (0.224 mmol) of 1,2-bis(diphenylphosphino)benzene, $(Ph_2P)_2$-1,2-C_6H_4 (DPPBz) is added to a 100 mL Schlenk flask and dissolved in 20 mL of CH_2Cl_2. To this solution 1 equivalent of [Co(acac)(dioxane)$_4$](BF_4)•(dioxane)$_x$ (x ≈ 1-4) complex (0.224 mmol) dissolved in 20 mL of acetone is added slowly via pipet while stirring. The resulting solution is stirred for 30 mins and the solvent is removed under vacuum. This yields 0.081 g of a dark red powder [Co(acac)(DPPBz)]BF_4 with an isolated yield of 52% (typical yields = 50-60%). If the resulting solid is sticky (due to too much dioxane from the Co(II) starting material) then the material is dissolved in CH_2Cl_2 and the solvent removed under vacuum to yield a dry powdered material. ^{1}H NMR (400 MHz, acetone-d$_6$, 24°C) δ = 32.2 ($\Delta v_{1/2}$ = 661 Hz), 16.0 ($\Delta v_{1/2}$ = 61 Hz), 9.80 ($\Delta v_{1/2}$ = 45.7 Hz), 9.42 ($\Delta v_{1/2}$ = 101 Hz), 8.19 ($\Delta v_{1/2}$ = 18 Hz), 8.05 ($\Delta v_{1/2}$ = 25 Hz), 7.80 ($\Delta v_{1/2}$ = 21.5 Hz), 7.70 ($\Delta v_{1/2}$ = 21 Hz), 7.62 ($\Delta v_{1/2}$ = 17.7 Hz), 7.42 ($\Delta v_{1/2}$ = 20 Hz), 7.31 ($\Delta v_{1/2}$ = 21.2 Hz), 6.77 ($\Delta v_{1/2}$ = 45 Hz), 6.38 ($\Delta v_{1/2}$ = 12.1 Hz), 5.57 ($\Delta v_{1/2}$ = 54 Hz), 5.21 ($\Delta v_{1/2}$ = 14.9 Hz), 4.62 ($\Delta v_{1/2}$ = 73 Hz), 4.13 ($\Delta v_{1/2}$ = 35 Hz), 3.69 ($\Delta v_{1/2}$ = 14 Hz, dioxane), 2.40 ($\Delta v_{1/2}$ = 11.2 Hz), 2.11 ($\Delta v_{1/2}$ = 9.1 Hz), 2.00 ($\Delta v_{1/2}$ = 32 Hz), 1.33 ($\Delta v_{1/2}$ = 15.2 Hz), 0.21 ($\Delta v_{1/2}$ = 25 Hz), and -6.1 ppm ($\Delta v_{1/2}$ = 266 Hz). IR (cm^{-1}, diamond ATR cell, water present due to running sample in air, see Figure S1): 3476.9 (br), 3057.6 (w), 2978.4 (br, w), 2920.2 (w), 2892.8 (w), 1712.6 (w), 1556.5 (s), 1519.5 (vs), 1435.2(m), 1366.8 (s), 1265.4 (w), 1093.8 (sh, m), 1052.3 (vs), 1022.5 (vs), 997.9 (sh, s), 931.5 (sh, m), 871.0 (w), 748.2 (w), 692.8 (m). High resolution mass spectrum

($C_{35}H_{31}CoO_2P_2$): 604.1131 (calc), 604.11254 (exp), excellent isotopic abundance match (Figure S7).

[Co(acac)(dppe)]BF_4:

0.100g (0.251 mmol) of 1,2-bis(diphenylphosphino)ethane, $Ph_2PCH_2CH_2PPh_2$ (dppe) is added to a 100 mL Schlenk flask and dissolved in 20 mL of CH_2Cl_2. To this solution 1 equivalent of [Co(acac)(dioxane)$_4$](BF_4)•(dioxane)$_x$ (x ≈ 1-4) complex (0.251 mmol) dissolved in 20 mL of acetone is added slowly via pipet while stirring. The resulting solution is stirred for 30 mins and the solvent is removed under vacuum. This yields 0.087 g of dark brown powder [Co(acac)(dppe)]BF_4 with an isolated yield of 54% (typical yields = 50-60%). If the resulting solid is sticky (due to too much dioxane from the Co(II) starting material) then the material is dissolved in CH_2Cl_2 and the solvent removed under vacuum to yield a dry powdered material. ^{1}H NMR (400 MHz, acetone-d$_6$, 24°C) δ = 9.35 ($\Delta v_{1/2}$ = 269 Hz), 8.20 ($\Delta v_{1/2}$ = 22 Hz), 7.60 ($\Delta v_{1/2}$ = 37 Hz), 7.50 ($\Delta v_{1/2}$ = 35 Hz), 7.46 ($\Delta v_{1/2}$ = 24 Hz), 7.38 ($\Delta v_{1/2}$ = 27 Hz), 7.03 ($\Delta v_{1/2}$ = 21 Hz), 6.33 ($\Delta v_{1/2}$ = 12 Hz), 3.58 ($\Delta v_{1/2}$ = 10 Hz, dioxane), 2.36 ($\Delta v_{1/2}$ = 8.5 Hz), 2.19 ($\Delta v_{1/2}$ = 6.3 Hz), 1.40 ($\Delta v_{1/2}$ = 7.1 Hz), and -6.80 ($\Delta v_{1/2}$ = 856 Hz). IR (cm^{-1}, diamond ATR cell, water present due to running sample in air, see Figure S1): 3480.0 (br, w), 3055.8 (w), 2917.2 (w), 2852.8 (w), 1556.0 (s), 1519.4 (vs), 1483.4(w), 1435.0 (s), 1363.4 (s), 1284.2 (w), 1263.5 (br, w), 1189.7 (w), 1165.6 (w), 1093.8 (sh, s), 1050.9 (vs), 1024.4 (sh, s), 996.4 (sh, s), 930.0 (w), 872.4 (w), 811.9 (w), 745.5 (m), 692.4 (s). High resolution mass spectrum ($C_{31}H_{31}CoO_2P_2$): 556.1126 (calc), 556.11277 (exp), excellent isotopic abundance match (Figure S8). Elemental analysis for $C_{31}H_{31}BCoF_4O_2P_2$: Calculated %C: 57.88, %H: 4.86. Found: %C: 57.60, %H: 5.08.

[Co(acac)(depe)]BF_4:

0.100 g (0.485 mmol) of 1,2-bis(diethylphosphino)ethane, $Et_2PCH_2CH_2PEt_2$ (depe), is added to a 100 mL Schlenk flask and dissolved in 20 mL of CH_2Cl_2. To this solution one equivalent of [Co(acac)(dioxane)$_4$](BF_4)•(dioxane)$_x$ (x ≈ 1-4) complex (0.485 mmol) dissolved in 20 mL of acetone is added slowly via pipet while stirring. The resulting solution is stirred for 30 mins and the solvent is removed under vacuum. This yields 0.127 g of red powder [Co(acac)(depe)]BF_4 with an isolated yield of 58% (typical yields = 50-65%). If the resulting solid is sticky (due to too much dioxane from the Co(II) starting material) then the material is dissolved in CH_2Cl_2 and the solvent removed under vacuum to yield a power material. ^{1}H NMR (400 MHz, acetone-d$_6$, 24°C) δ = 6.37 ($\Delta v_{1/2}$ = 3.8 Hz), 3.68 ($\Delta v_{1/2}$ = 7.0 Hz, dioxane), 2.53 ($\Delta v_{1/2}$ = 31 Hz), 2.38 ($\Delta v_{1/2}$ = 6.8 Hz), 2.24 ($\Delta v_{1/2}$ = 12 Hz), 2.06 ($\Delta v_{1/2}$ = 11 Hz), 1.87 ($\Delta v_{1/2}$ = 3.7 Hz), 1.46,1.43,1.38,1.36,1.34,1.24 (overlapping signals, $\Delta v_{1/2}$ = 76 Hz), and -1.32 ppm ($\Delta v_{1/2}$ = 201 Hz). IR (cm^{-1}, diamond ATR cell, water present due to running sample in air, see Figure S1): 3515.8 (br, m), 2976.9 (w), 2950.3 (br, w), 2889.1 (w), 1555.3 (s), 1521.1 (vs), 1431.7 (sh, w), 1364.8 (s), 1267.4 (m), 1051.4 (vs), 1017.5 (vs), 931.2 (m), 872.3 (w), 763.7 (br, w).

[Co(acac)(DEPBz)]BF_4:

0.100 g (0.393 mmol) of 1,2-bis(diethylphosphino)benzene, (Et_2P)2-1,2-C_6H_4 (DEPBz), is added to a 100 mL Schlenk flask and dissolved in 20 mL of CH_2Cl_2. To this solution 1 equivalent of [Co(acac)(dioxane)$_4$](BF_4)•(dioxane)$_x$ (x ≈ 1-4) complex (0.393 mmol) dissolved in 20 mL of acetone is added slowly via pipet while stirring. The resulting solution is stirred for 30

mins and the solvent is removed under vacuum. This yields 0.110 g of a dark brown powder [Co(acac)(DEPBz)]BF_4 with an isolated yield of 56% (typical yields = 50-65%). If the resulting solid is sticky (due to too much dioxane from the Co(II) starting material) then the material is dissolved in CH_2Cl_2 and the solvent removed under vacuum to yield a dry powered material. ^1H NMR (400 MHz, acetone-d_6, 24°C) δ = 8.60 ($\Delta v_{1/2}$ = 17 Hz), 8.4 ($\Delta v_{1/2}$ = 10 Hz), 8.24 ($\Delta v_{1/2}$ = 16 Hz), 8.12 ($\Delta v_{1/2}$ = 18 Hz), 8.06 ($\Delta v_{1/2}$ = 14 Hz), 7.88 ($\Delta v_{1/2}$ = 23 Hz), 6.90 ($\Delta v_{1/2}$ = 24 Hz), 6.58 ($\Delta v_{1/2}$ = 50 Hz), 6.41 ($\Delta v_{1/2}$ = 7.4 Hz), 6.06 ($\Delta v_{1/2}$ = 5.8 Hz), 5.73 ($\Delta v_{1/2}$ = 7.7 Hz), 5.59 ($\Delta v_{1/2}$ = 23 Hz), 4.37 ($\Delta v_{1/2}$ = 24 Hz), 3.72 ($\Delta v_{1/2}$ = 9.8 Hz, dioxane), 3.52 ($\Delta v_{1/2}$ = 11 Hz), 3.51 ($\Delta v_{1/2}$ = 6.8 Hz), 3.36 ($\Delta v_{1/2}$ = 26 Hz), 3.08 ($\Delta v_{1/2}$ = 28 Hz), 2.43 ($\Delta v_{1/2}$ = 5.3 Hz), 2.35 ($\Delta v_{1/2}$ = 9.4 Hz), 2.11 ($\Delta v_{1/2}$ = 7.8 Hz), 1.86 ($\Delta v_{1/2}$ = 6.5 Hz), 1.63 ($\Delta v_{1/2}$ = 21 Hz), 1.55, 1.51, 1.49 (overlapping signals, $\Delta v_{1/2}$ = 32 Hz), 1.39, 1.36, 1.34, 1.32 (overlapping signals, $\Delta v_{1/2}$ = 38 Hz), 1.22, 1.21, 1.19, 1.14 (overlapping signals, $\Delta v_{1/2}$ = 55 Hz), 1.07, 1.04, and 0.97(overlapping signals, $\Delta v_{1/2}$ = 43 Hz). IR (cm^{-1}, diamond ATR cell, water present due to running sample in air, some dioxane present, see Figure S2): 3500.3 (br, m), 3232.1 (sh, br, m), 2975.1 (w), 2932.6 (sh, br, w), 2887.9 (w), 1696.3 (w), 1640.0 (w), 1571.5 (m), 1521.3 (vs), 1366.1 (s), 1264.8 (m), 1045.3 (sh, vs), 1016.2 (vs), 929.7 (sh, m), 869.6 (m), 763.0 (w).

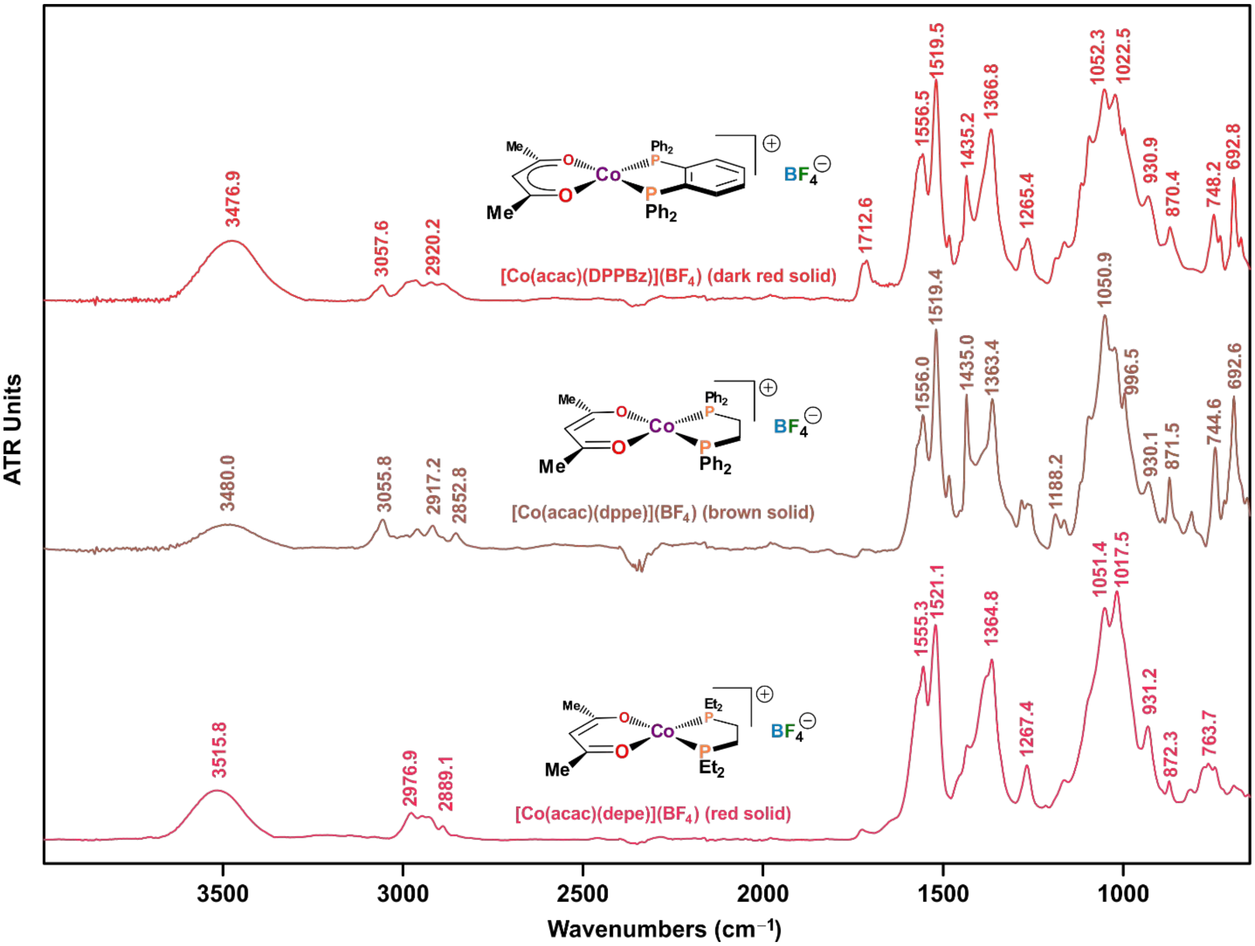

Fig. S1. FT-IR spectra of [Co(acac)(DPPBz)](BF_4), [Co(acac)(dppe)](BF_4), and [Co(acac)(depe)](BF_4).

FT-IR spectra of [Co(acac)(DPPBz)](BF_4), [Co(acac)(dppe)](BF_4), and [Co(acac)(depe)](BF_4). Samples were run in air and some atmospheric water absorption has occurred for the cobalt complexes. FT-IR spectra are the same as those run under N_2, except for the increased water bands. Reprinted with permission from Ref. 1.

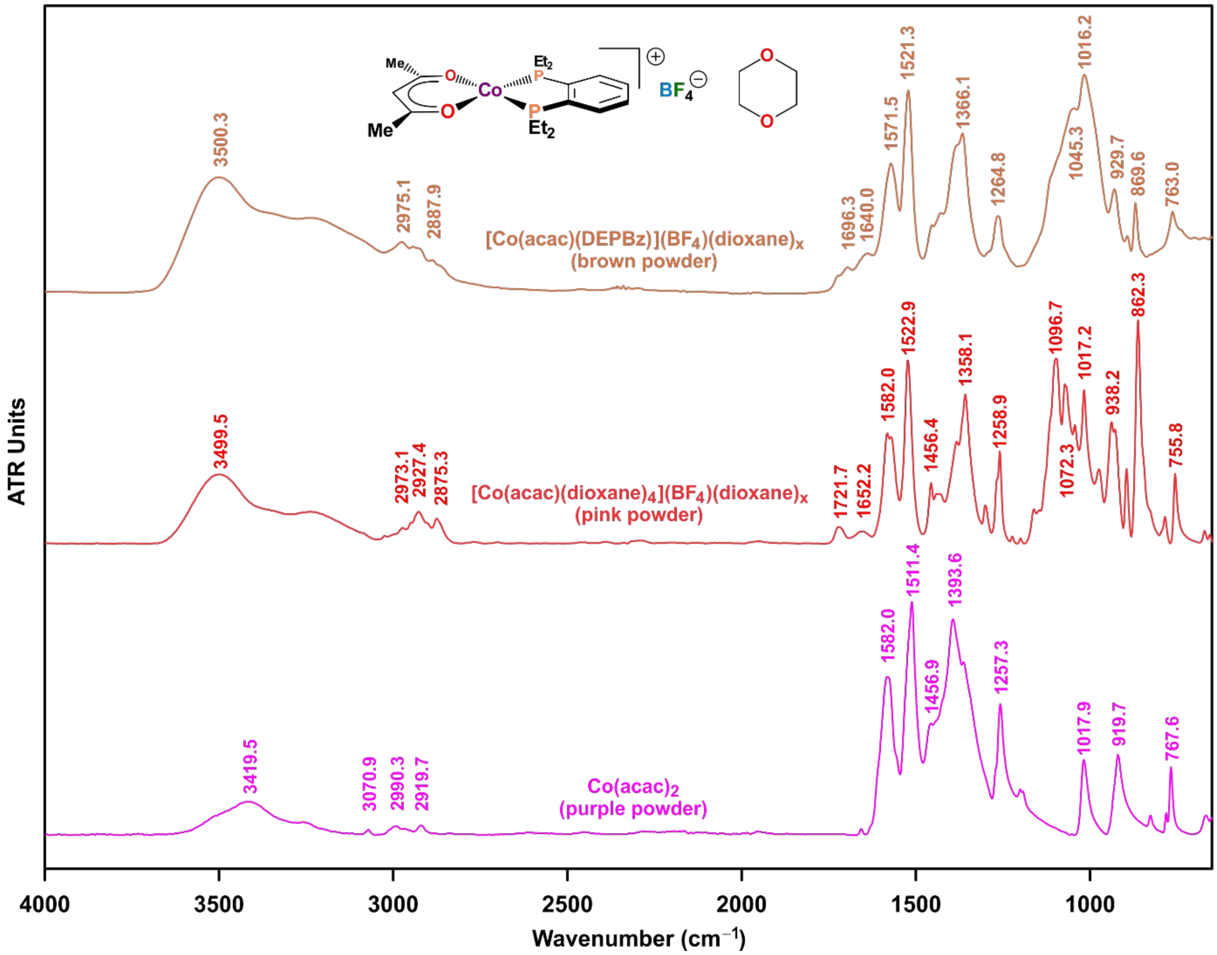

Fig. S2. FT-IR spectra of [Co(acac)(DEPBz)](BF_4) and starting materials.

FT-IR spectra of Co(acac)$_2$ (bottom, purple), [Co(acac)(dioxane)$_4$](BF_4)(dioxane)$_x$ (middle, red), and [Co(acac)(DEPBz)](BF_4)(dioxane)$_x$ catalyst precursor (top, brown). Samples were run in air and some atmospheric water absorption has occurred for both cobalt complexes. FT-IR spectra are the same as those run under N_2, except for the increased water bands. Reprinted with permission from Ref. 1.

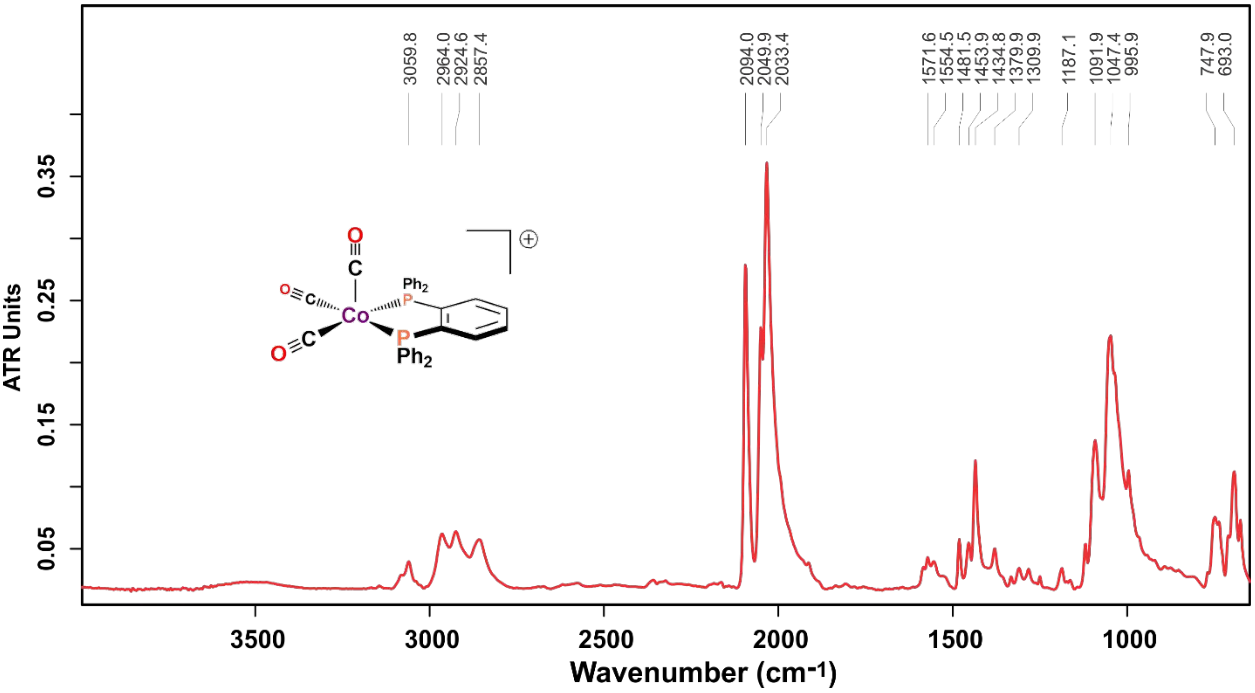

Figure S3. FT-IR spectrum of $[Co(CO)_3(DPPBz)](BF_4)$.

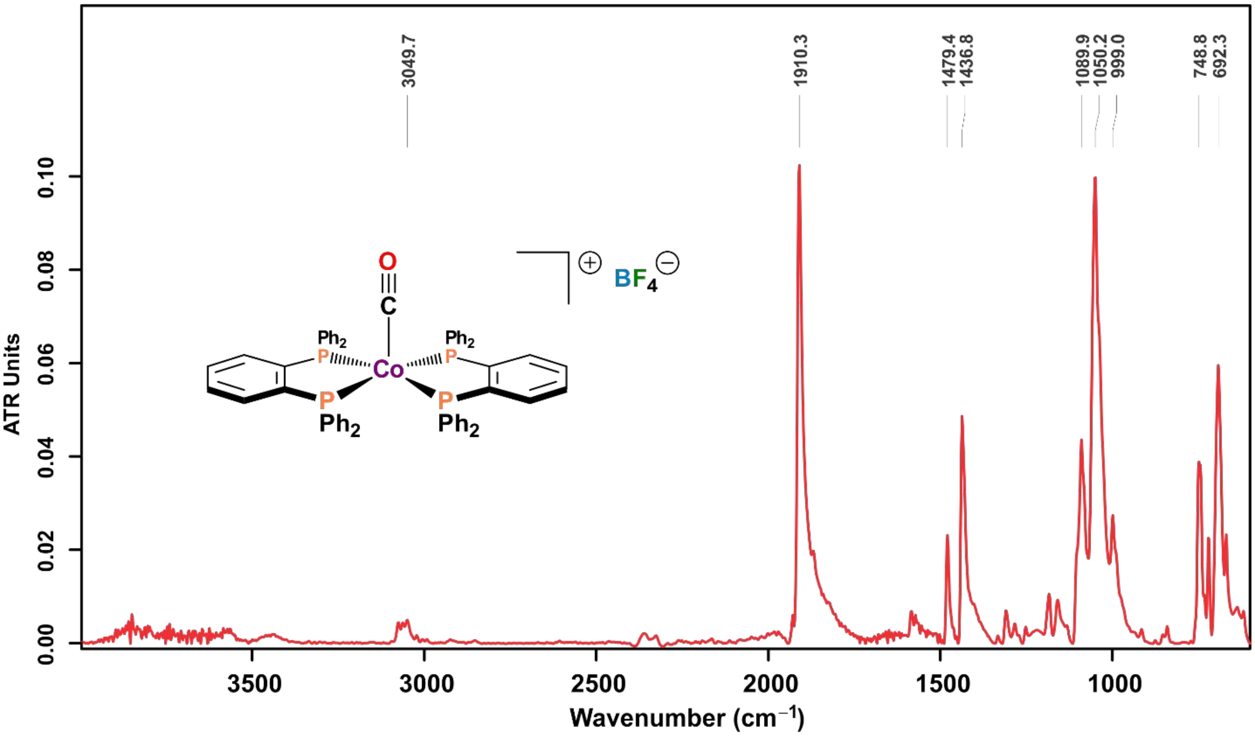

Figure S4. FT-IR spectrum of [Co(CO)(DPPBz)$_2$](BF$_4$).

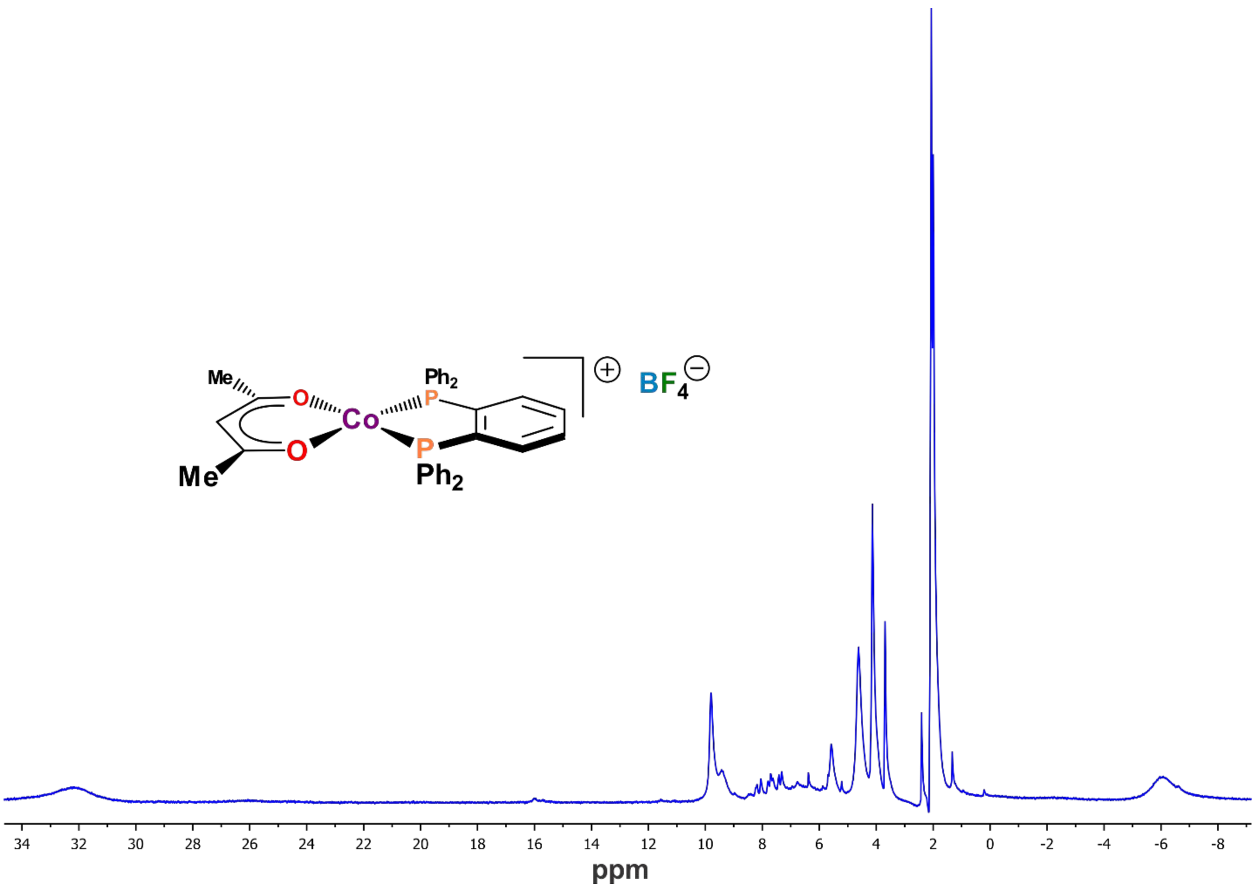

Fig. S5. ^{1}**H NMR spectrum of [Co(acac)(DPPBz)](BF_4) in acetone-d_6.**

Acetone solvent is contributing to the strong acac methyl group peak at 2 ppm. Dioxane solvent at 3.5 ppm is also present from the starting material. Reprinted with permission from Ref. 1.

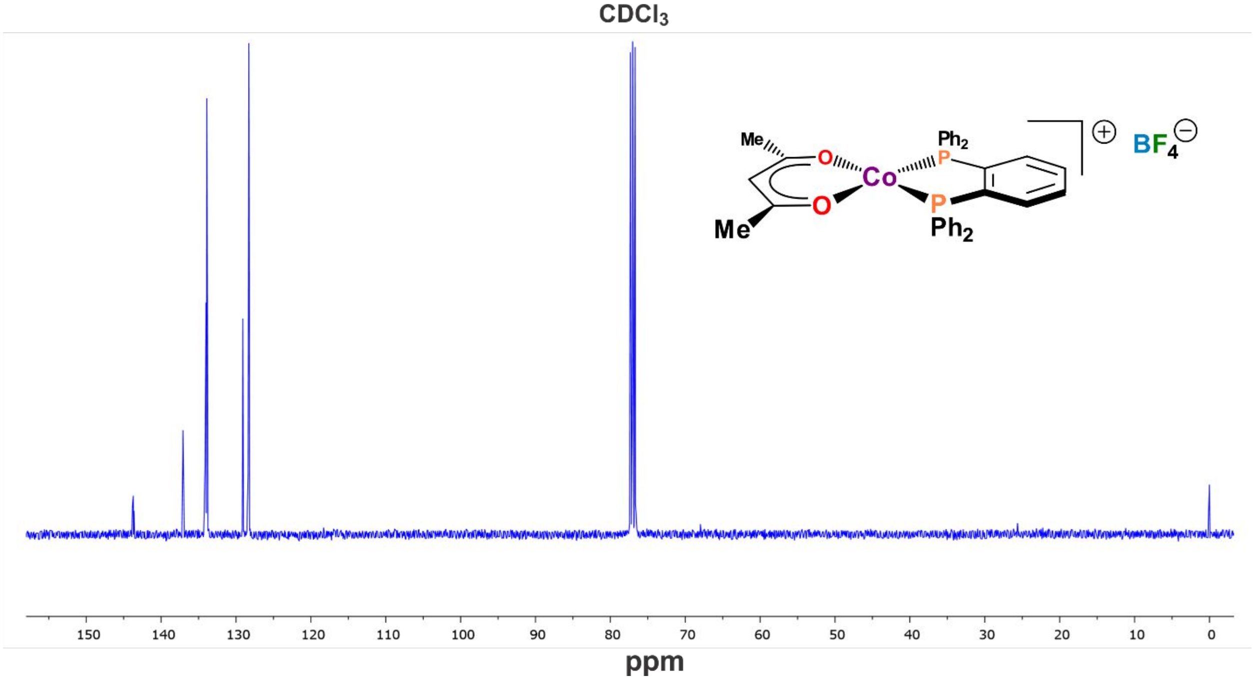

Fig. S6. ^{13}C NMR spectrum of [Co(acac)(DPPBz)](BF_4) in $CDCl_3$. Reprinted with permission from Ref. 1.

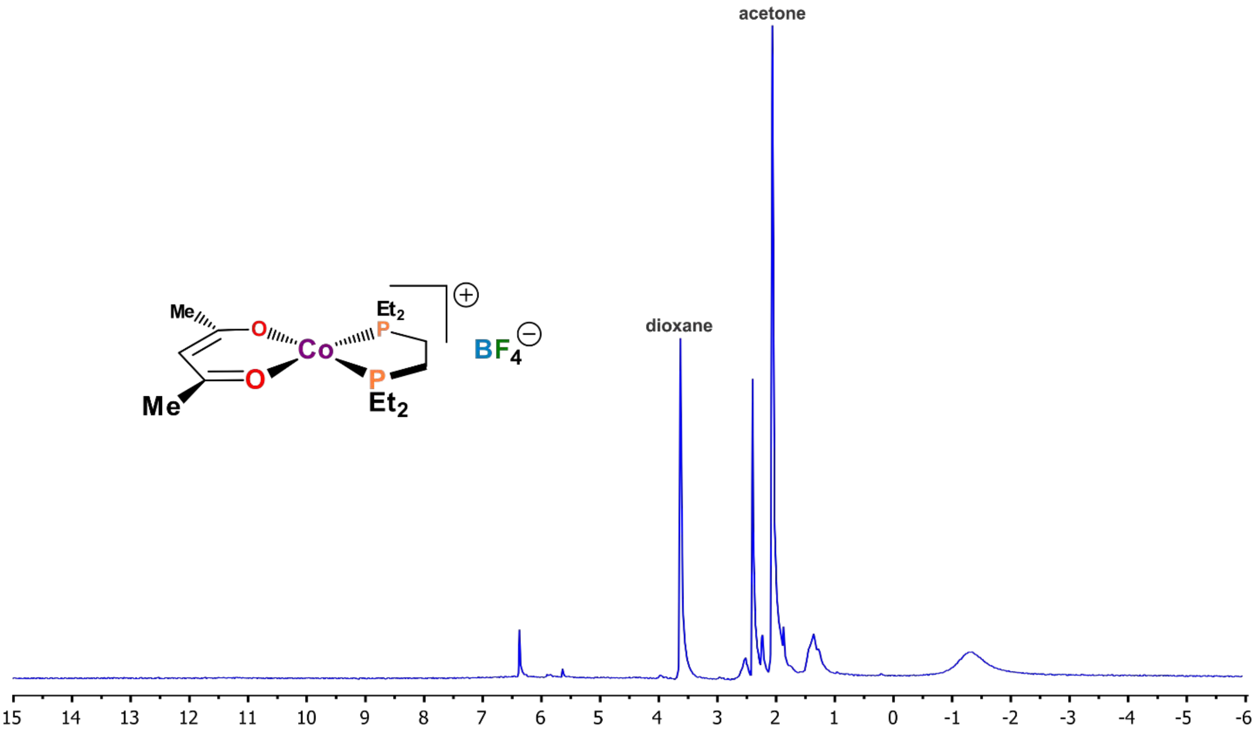

Fig. S7. ^{1}H NMR spectrum of [Co(acac)(depe)](BF_4) in acetone-d_6.
Acetone solvent is contributing to the acac methyl group peak at 2 ppm. Dioxane solvent is also present from the starting material. Reprinted with permission from Ref. 1.

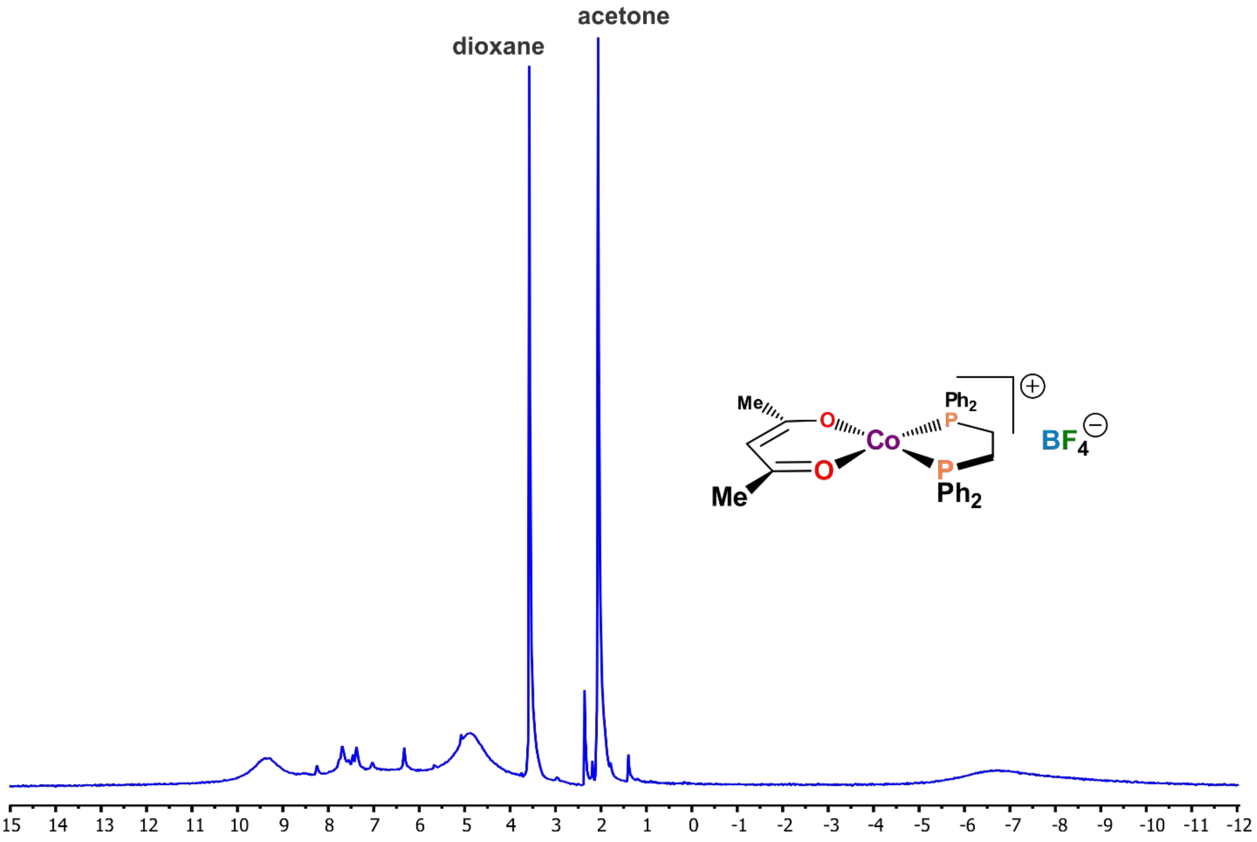

Fig. S8. ^{1}**H NMR spectrum of [Co(acac)(dppe)](BF_4) in acetone-d_6.**

Acetone solvent is contributing to the acac methyl group peak at 2 ppm. Dioxane solvent is also present from the starting material. Reprinted with permission from Ref. 1.

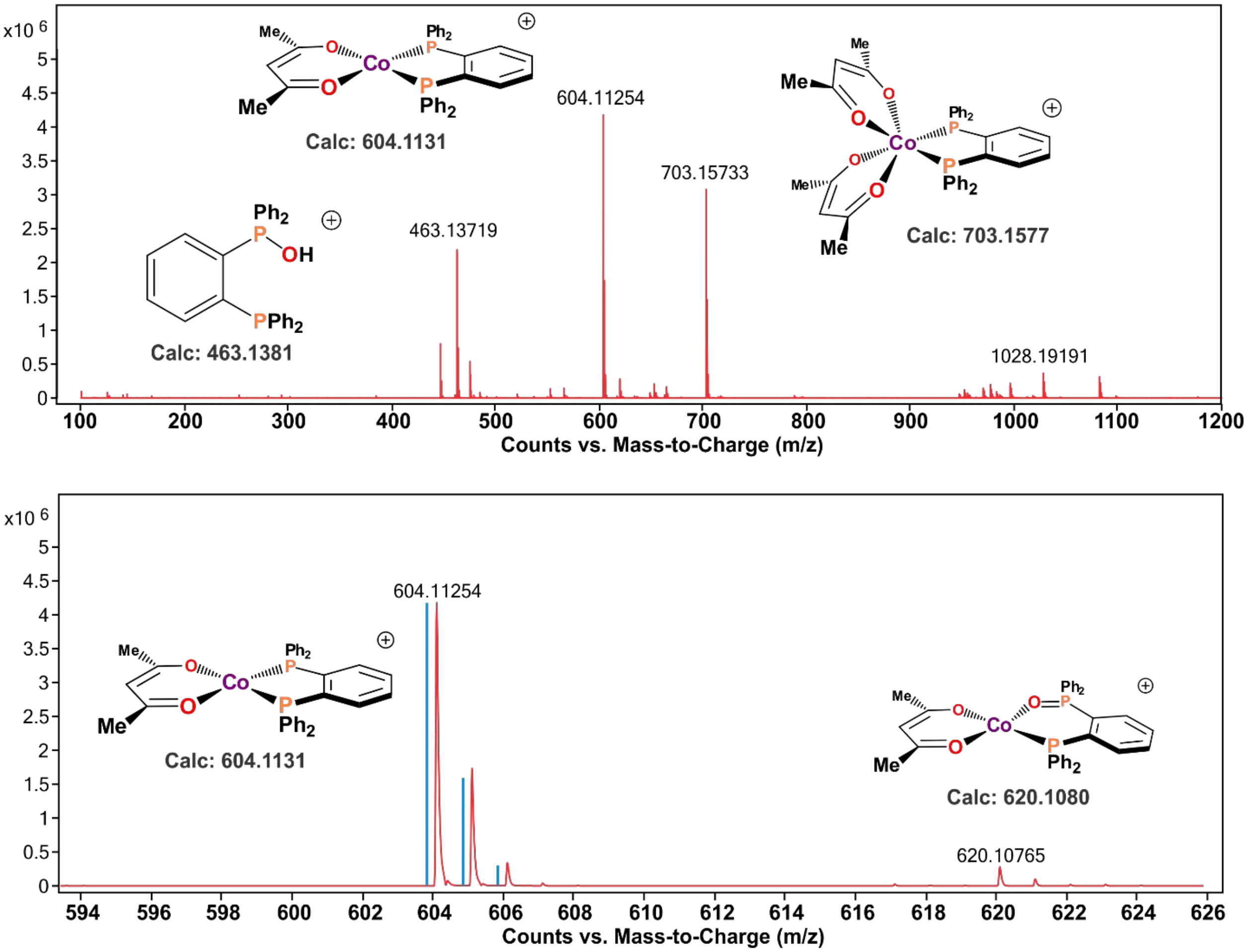

Fig. S9. High resolution electrospray mass spectrum of [Co(acac)(DPPBz)]$^+$.

Top spectrum shows the full mass range and peaks assigned for the high resolution electrospray mass spectrum on [Co(acac)(DPPBz)](BF_4), DPPBz = (Ph_2P)$_2$-1,2-C_6H_4. The major species are assigned with their calculated exact masses with the experimental masses for the parent peak listed. The bottom spectrum is an expanded region that shows the calculated isotopic distribution intensity pattern in blue offset from the red experimental peaks. The samples were run in air and with a 60:40 solvent system of acetonitrile and 0.1 % formic acid/water, which formed the side products that are present. Reprinted with permission from Ref. 1.

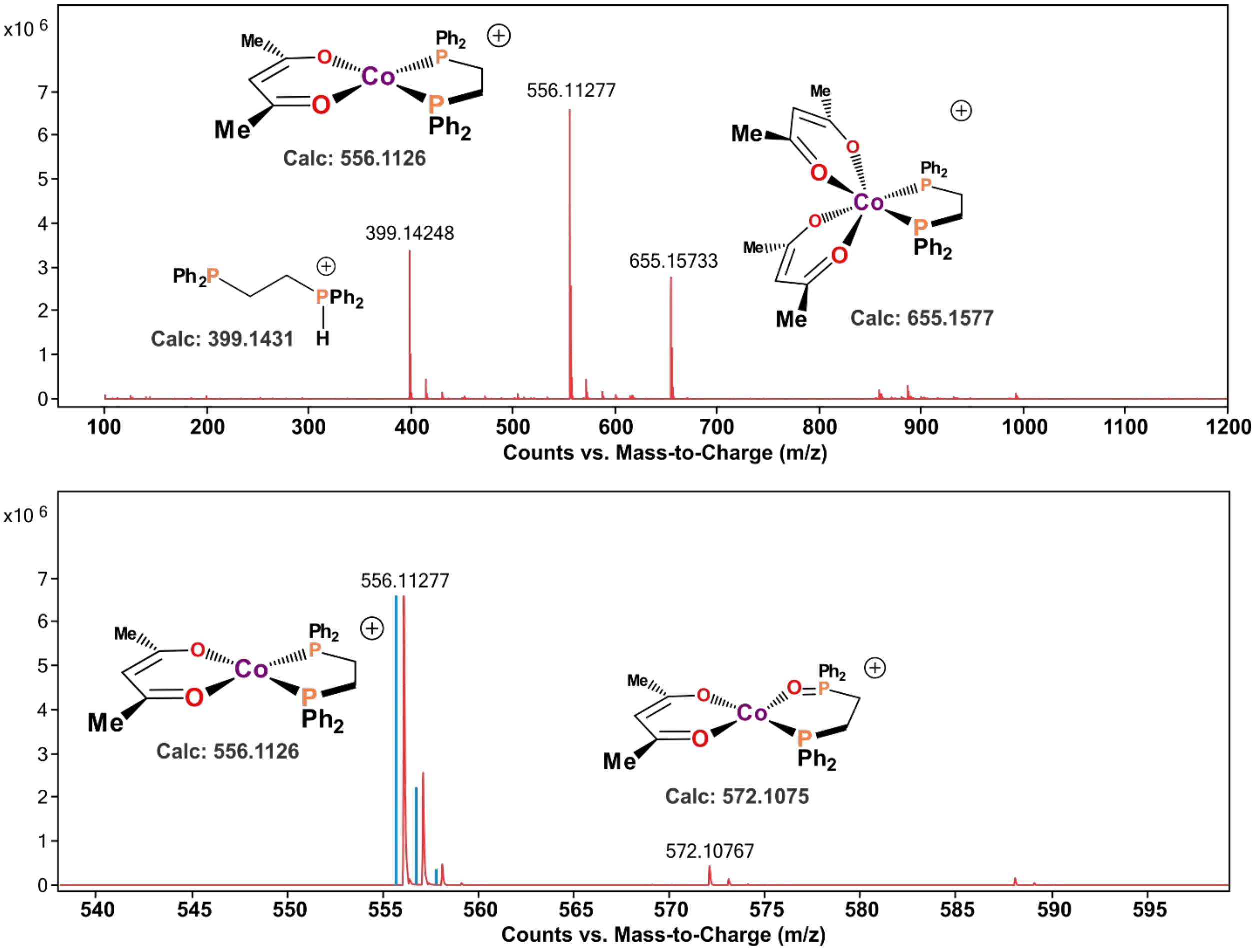

Fig. S10. High resolution electrospray mass spectrum of [Co(acac)(dppe)]$^+$.

Top spectrum shows the full mass range and peaks assigned for the high resolution electrospray mass spectrum on [Co(acac)(dppe)](BF_4), dppe = $Ph_2PCH_2CH_2PPh_2$. The major species are assigned with their calculated exact masses with the experimental masses for the parent peak listed. The bottom spectrum is an expanded region that shows the calculated isotopic distribution intensity pattern in blue offset from the red experimental peaks. The samples were run in air and with a 60:40 solvent system of acetonitrile and 0.1 % formic acid/water, which formed the side products that are present. Reprinted with permission from Ref. 1.

LSU Hydroformylation Experiments:

Safety Note: CO is toxic and our autoclave systems are located in a hood (two per hood). A CO detector is located at the front of each autoclave hood and by the computer connected to the Parr 4870 process controller that is near the hoods with the autoclaves.

Liquid alkenes used in the catalytic runs were degassed and passed down a short alumina column to remove peroxide impurities. The neutral alumina was prepped by completely drying it in the oven at 250°C overnight. The alumina was then placed under vacuum while still hot using a Schlenk flask. Once the alumina cooled it was transferred to an inert atmosphere (N_2) glove box used for reactions involving water and 10% water by weight added. The addition of water is exothermic and the alumina can get pretty hot. The alumina will also clump so it has to be shaken well to ensure an even mixture. 8-12% water works with 10% water typically used.

All runs reported, with the exception of the million turnover experiment, were performed under the conditions outlined below. The autoclave was first purged with nitrogen, then pressure was reduced under vacuum. After evacuation the alkene sample reservoir was sealed off from the rest of the autoclave. The alkene sample reservoir was charged with the desired amount of alkene via cannula using standard inert-atmosphere techniques. The catalyst precursor solution, which consist of the catalyst precursor and standard dissolved in a given solvent, was transferred from a sealed flask to the autoclave via cannula using standard inert-atmosphere techniques. The autoclave is brought to atmospheric pressure with N_2.

Next a flexible steel hose was purged with high purity 1:1 H_2:CO gas (premixed by the gas vendor in an aluminum tank), then attached to the closed alkene sample reservoir and the sample withdrawal arm (see figs. S11 & S12). The autoclave is pressurized via the sample withdrawal arm with H_2:CO. Pressures are monitored via an electronic pressure transducer connected to a Parr 4870 process controller. The sample withdrawal arm was then closed to reseal the autoclave, after which the autoclave was set to the desired reaction temperature. The top valve to the alkene sample reservoir is closed to isolate the alkene from active H_2:CO pressure. The autoclave was allowed to heat until the desire temperature was reached (typically 15-20 mins) after which the autoclave pressure was reduced to approximately 90% of the desired reaction pressure via the venting arm.

The reaction is initiated by opening the sealed autoclave to the alkene sample reservoir, which forces some of the hot catalyst solution into the alkene reservoir. This is immediately followed by opening the valve between the alkene sample reservoir and the gas feed line, which is at full operating pressure and forces all the alkene and catalyst mixture back into the autoclave. Finally the valve between the sample withdrawal arm and the gas feed line was opened. Our autoclave setup injects gas via two inlets into the catalyst solution to ensure optimum gas mixing and delivery.

Catalyst samples during a run are taken by closing the top and bottom valves on the sample withdrawal arm, followed by releasing the pressure via the side valve. Opening the bottom valve forces catalyst solution into the now low-pressure, small volume, sample withdrawal arm section. After closing the bottom valve, the side valve was opened to squirt a small amount of catalyst solution into a sample vial for GC/MS analysis. Afterwards, the top and bottom valves on the sample withdrawal arm are opened and the remainder of the trapped catalyst solution in the sample withdrawal arm is returned to the autoclave. This sampling procedure is safe even for high-pressure runs due to the limited volume in the sample withdrawal arm.

The million turn over experiment was run similarly to the standard conditions outlined above, except that the catalyst solution was loaded into the smaller sample reservoir arm and the autoclave was charged with the larger volume alkene-solvent mixture. The alkene-solvent mixture was heated under 1:1 H_2:CO pressure to operating conditions and the room temperature catalyst precursor solution was pressure injected into the alkene-solvent mixture.

Blank runs (alkene, solvent, no catalyst) are done regularly to check for proper autoclave cleaning. Although our research group also studies rhodium hydroformylation catalysts (mono- and bimetallic), we generally use separate autoclaves for the rhodium and cobalt reaction studies. The autoclaves are cleaned when needed with diamond paste to remove any decomposition products onto the autoclave interior surfaces (& stirrer). We do not use glass or Teflon liners in our autoclaves because they slow down the heating process to reach reaction temperature.

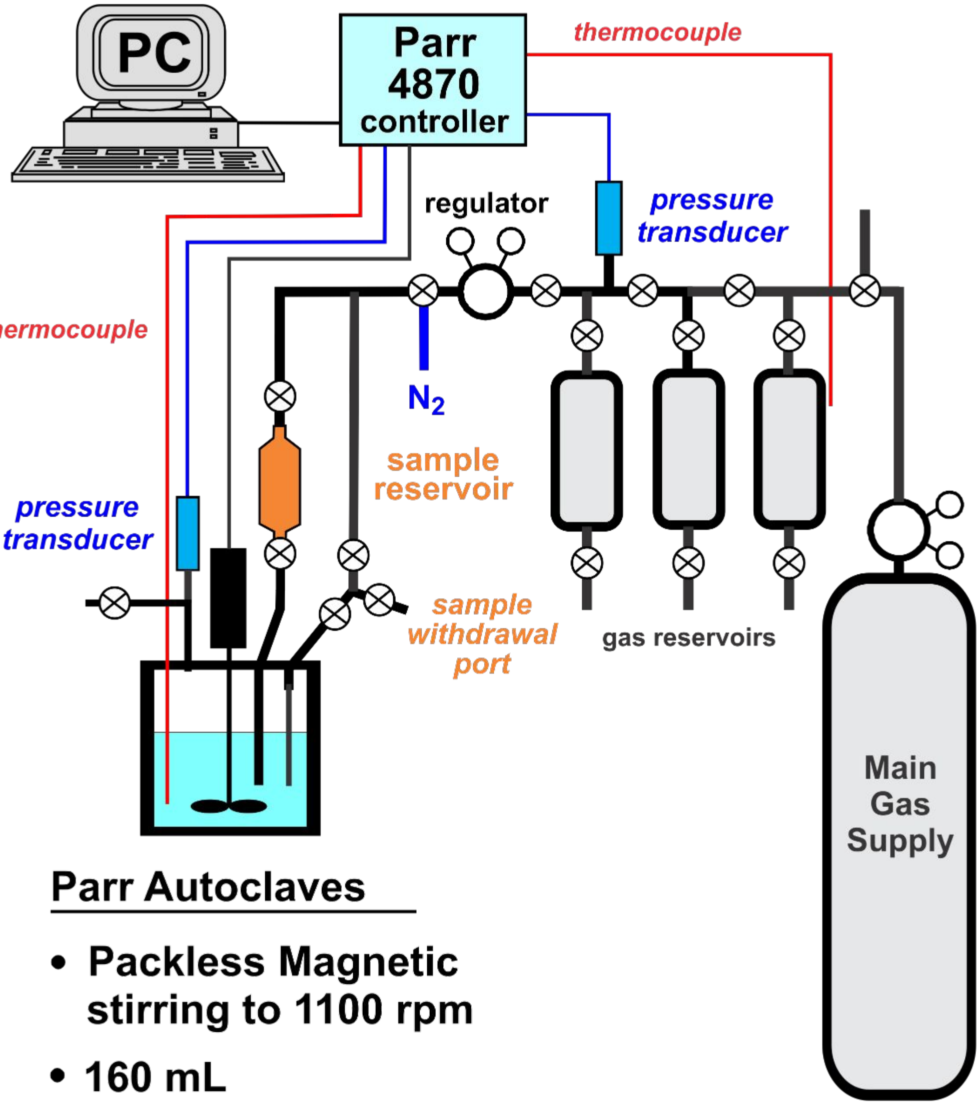

Fig. S11. Schematic drawing of one of the Parr autoclave systems.

Schematic drawing of one of the Parr autoclave systems used in this study. The Parr 4870 controller is connected to four such autoclaves that can each be independently operated and data collected on the Windows PC using SpecView v2.5 software. Catalyst precursor solution is typically loaded directly into the autoclave via cannula from a Schlenk flask after flushing with N_2 or H_2/CO gas. Alkene is loaded into the sample reservoir, which is then pressurized with the system. The sample reservoir can be heated, if needed, via the use of heat tape and a temperature controller. Once the alkene is pressure added to the autoclave at reaction conditions, the H_2/CO gas is introduced via both inlets into the autoclave solution. Samples can be removed for analysis during a catalysis run via the sample withdrawal port. By closing the top and bottom valves of the sample port, gas pressure can be released via the side valve. Opening the bottom valve allows catalyst solution to be pressure pushed into the sampling area. Closing the bottom valve and opening the side valve allows a small sample to be removed. The catalyst solution remaining can then be returned to the autoclave by re-opening the top and bottom valves. Reprinted with permission from Ref. 1.

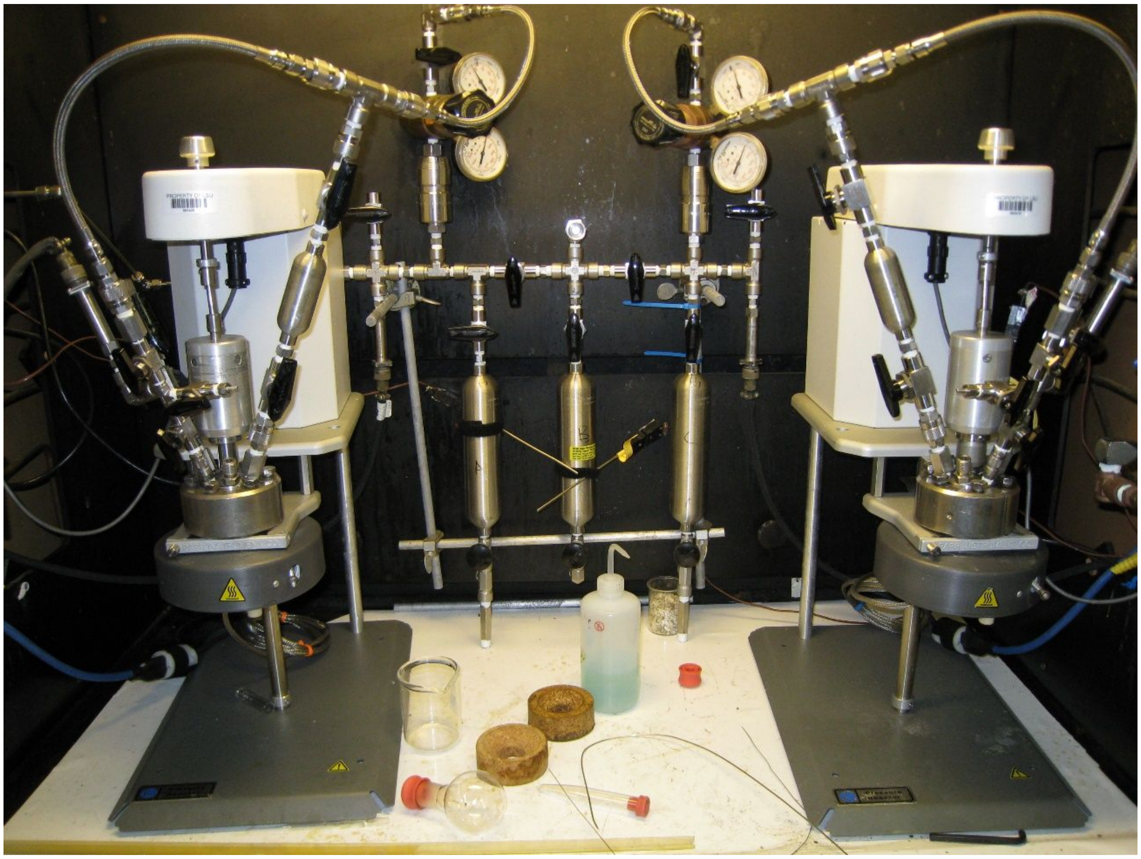

Fig. S12. Photograph of two of the Parr autoclave systems.

Photograph of two of the autoclave systems used for hydroformylation studies reported. Each autoclave has a design shown in Fig. S11 and share the three gas reservoirs that can be dedicated to one or the other autoclave system. Autoclaves are located in a fume hood with a CO detector mounted at the front of the hood. They are connected to a Parr 4870 process controller, along with two other autoclaves in a separate hood. Reprinted with permission from Ref. 1.

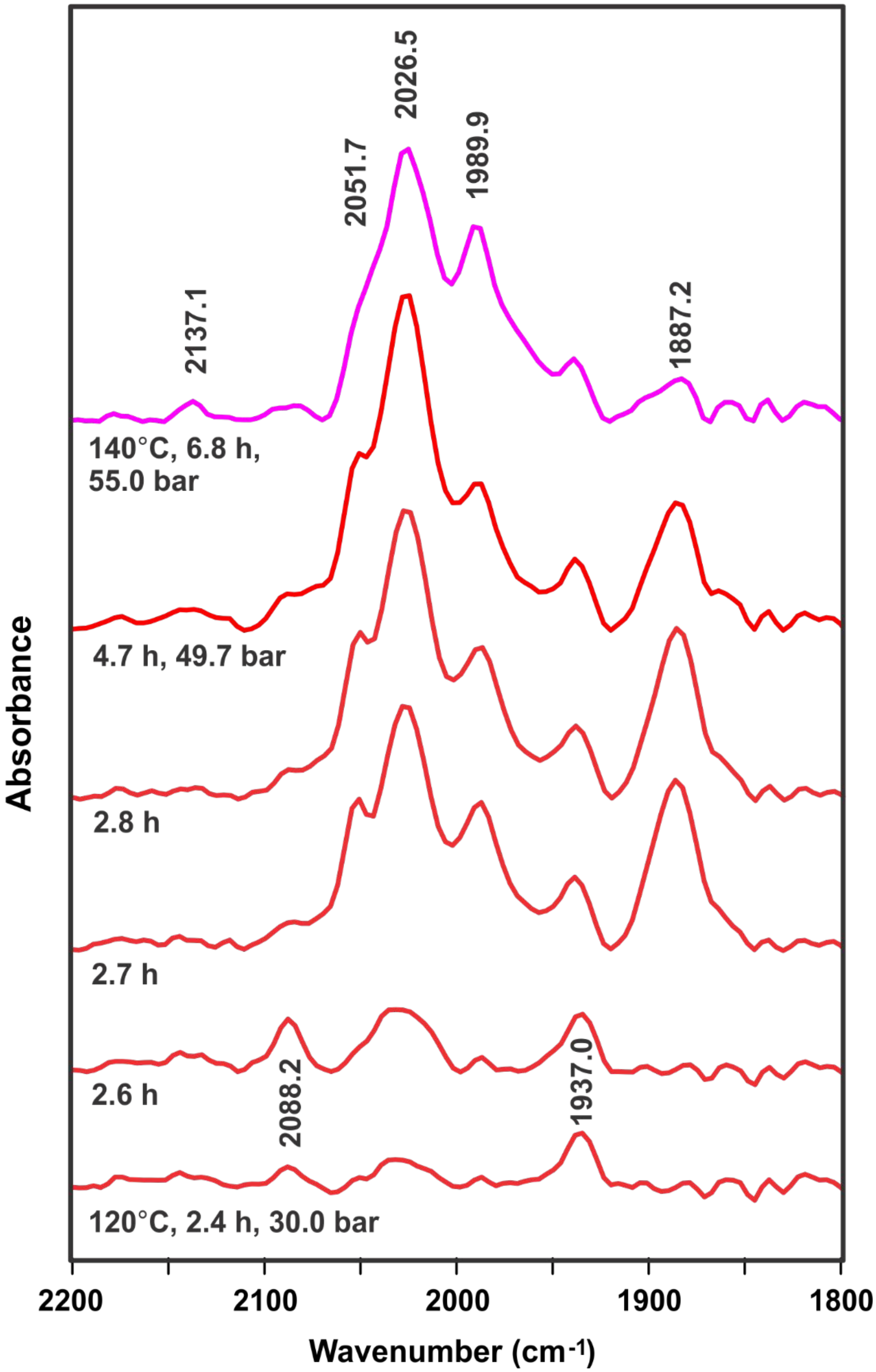

Fig. S13. ReactIR 101 hour study of [Co(acac)(DPPBz)](BF_4): 2.4 to 6.8 hrs.
10 mM concentration of [Co(acac)(DPPBz)](BF_4) in dimethoxytetraglyme, 1:1 H_2:CO. Subtracted solvent spectra taken at the given temperature.

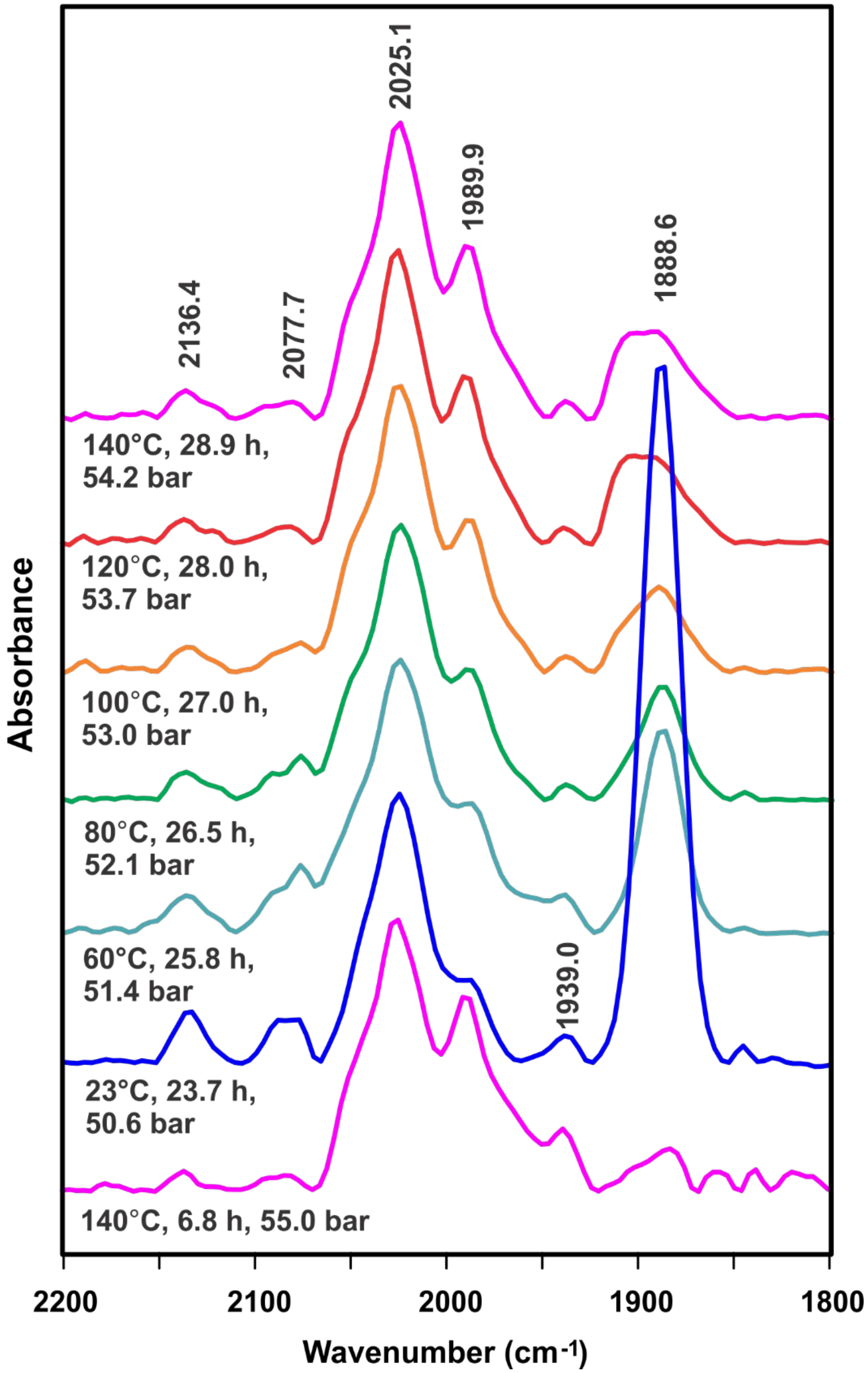

Fig. S14. ReactIR 101 hour study of [Co(acac)(DPPBz)](BF_4): 6.8 to 28.9 hrs.
The 1888 cm^{-1} bridging carbonyl band in the 23°C, 23.7 hr spectrum has a much higher intensity relative to the 2025 cm^{-1} terminal band for the proposed [Co_2(μ-CO)$_2$(CO)(DPPBz)$_2$](BF_4)$_2$ dimer complex. This is proposed to be due to some of the dimer complex crystallizing on the silicon ATR window after sitting at room temperature overnight with the bridging carbonyl bands oriented to selectively increase their intensity. Spectrum colors represent temperature.

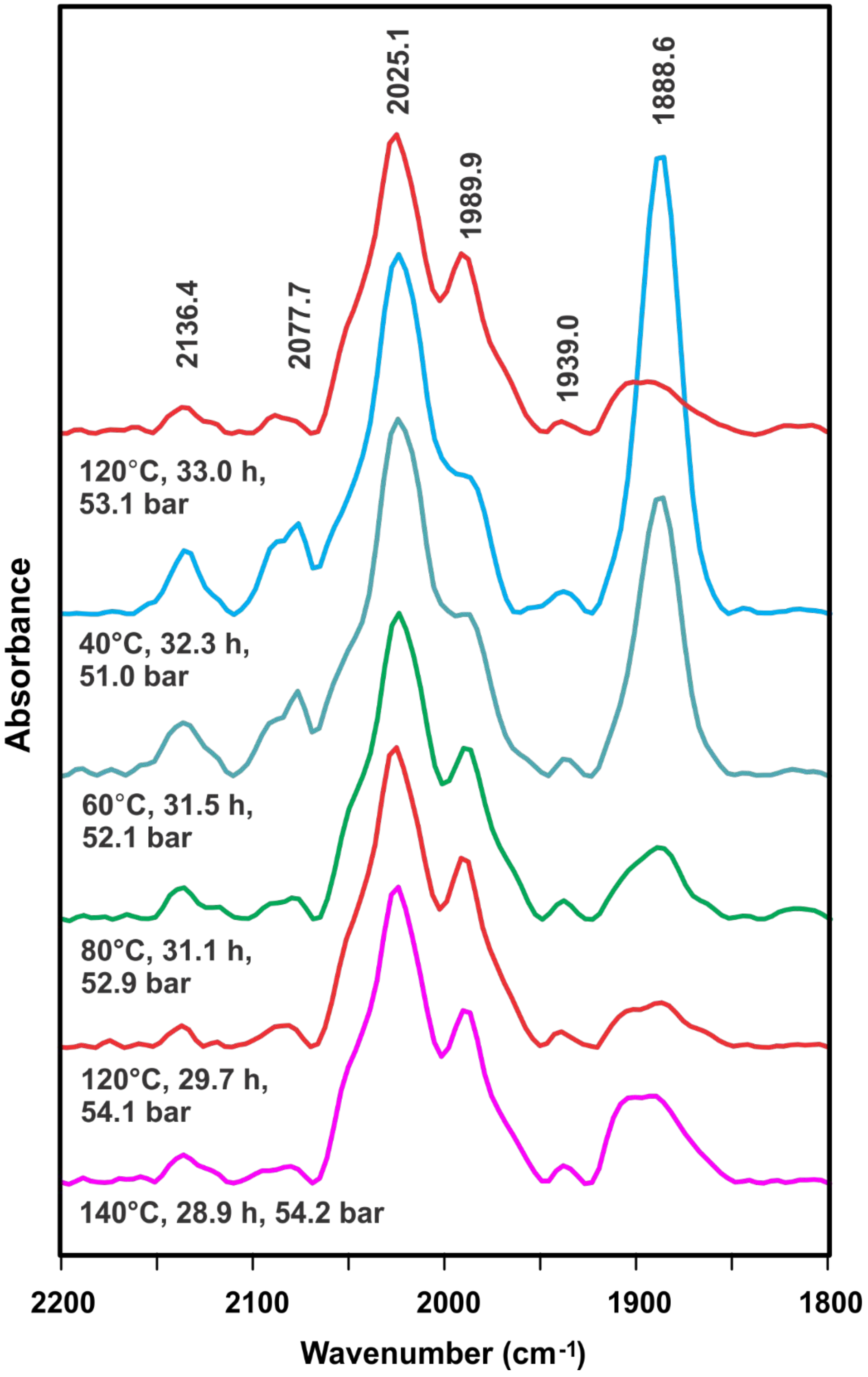

Fig. S15. ReactIR 101 hour study of [Co(acac)(DPPBz)](BF_4): 28.9 to 33.0 hrs. Spectrum colors represent temperature.

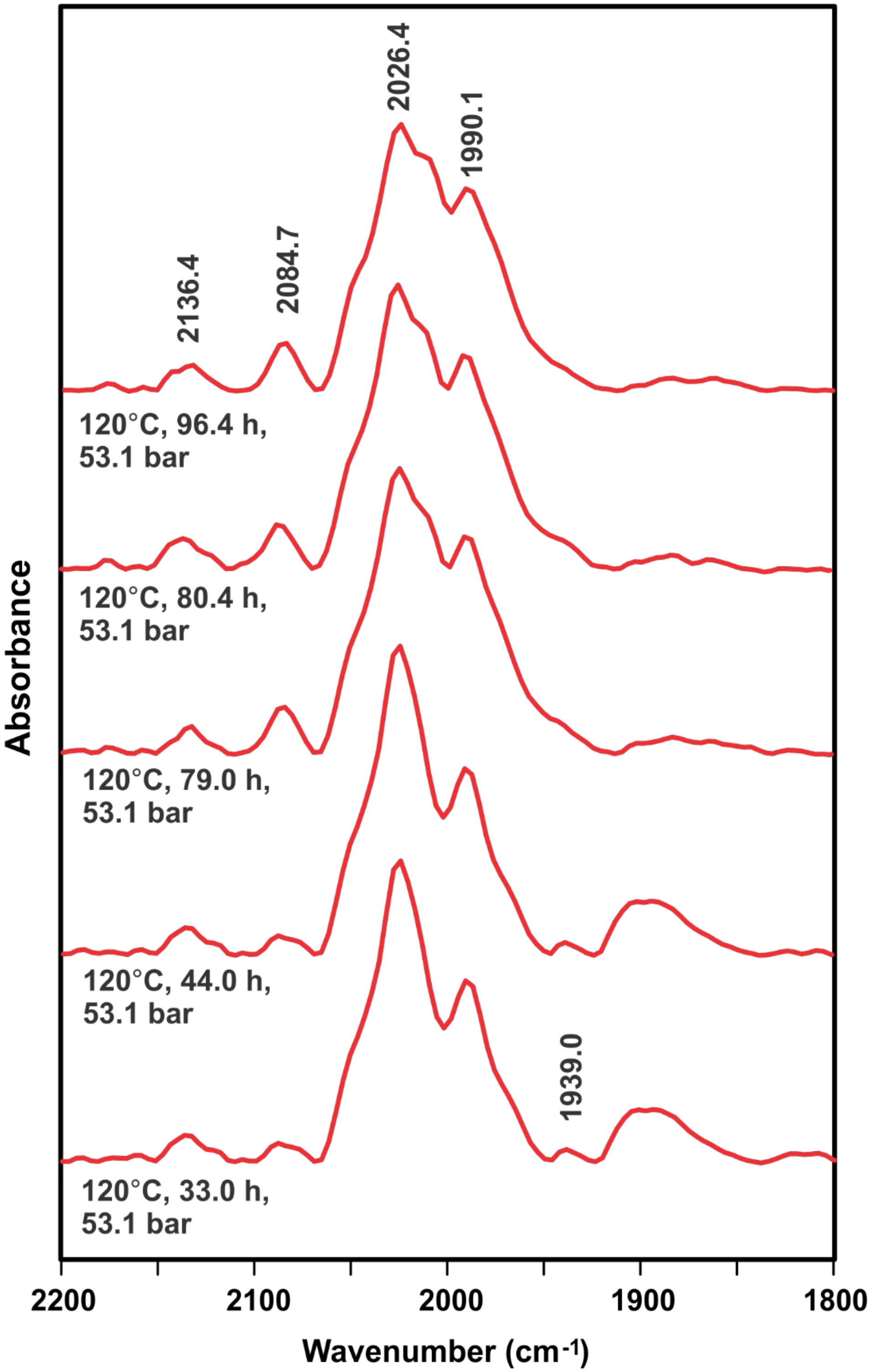

Fig. S16. ReactIR 101 hour study of [Co(acac)(DPPBz)](BF_4): 53.1 to 96.4 hrs.
Sample keep at 120°C during this series of spectra to test for catalyst stability. Reprinted with permission from Ref. 1.

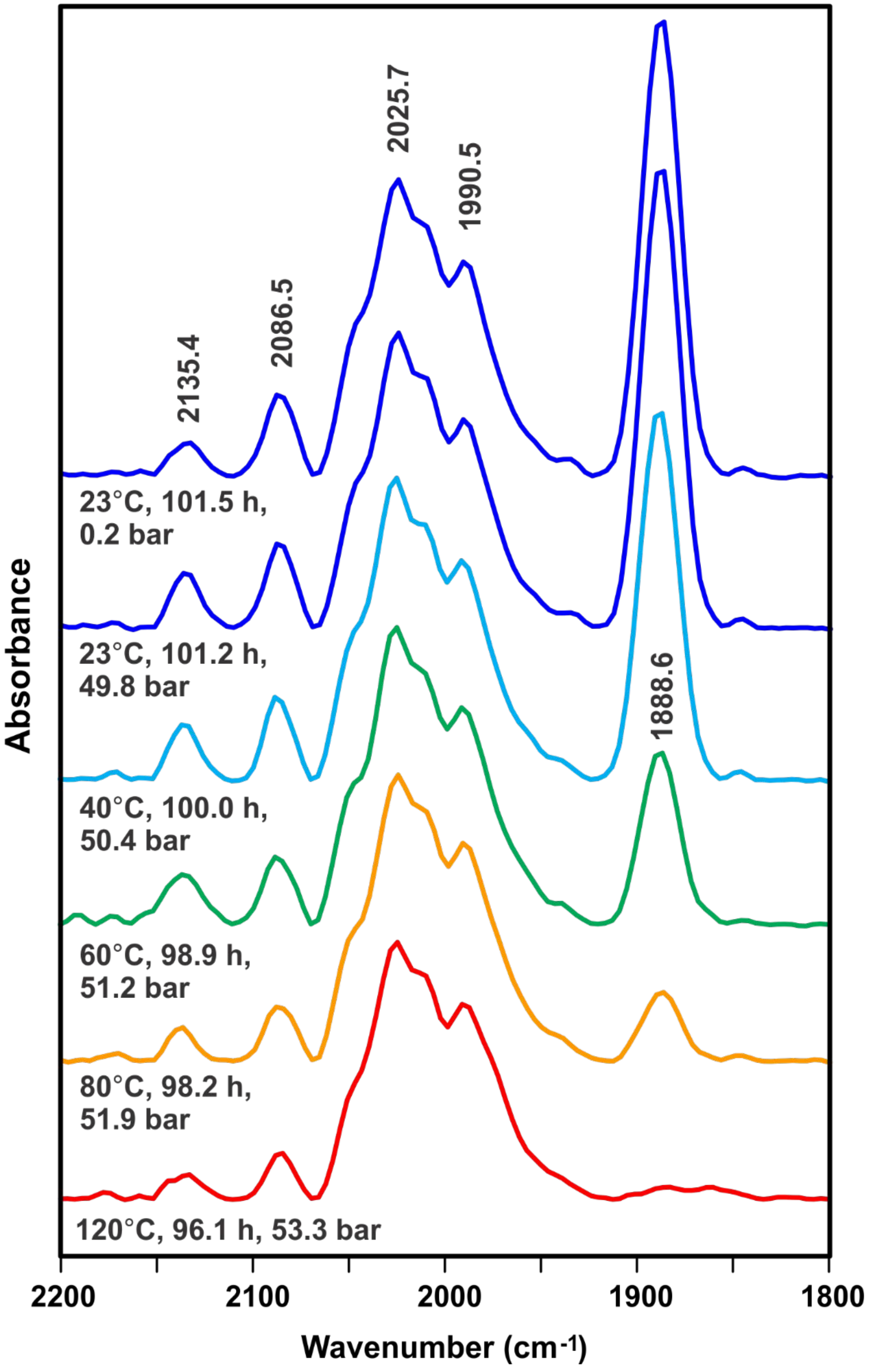

Fig. S17. ReactIR 101 hour study of [Co(acac)(DPPBz)](BF_4): 96.1 to 101.5 hrs. Spectrum colors represent temperature.

Crystallographic Structure Determinations

Intensity data were collected at low temperature on a Bruker Kappa Apex-II DUO CCD diffractometer fitted with an Oxford Cryostream chiller. Radiation was MoKα, from a fine-focus sealed tube with Triumph curved graphite monochromator. Data reduction included absorption corrections by the multiscan method using SADABS. The structures were determined by direct methods and difference Fourier techniques and refined by full-matrix least squares, using SHELXL. All non-hydrogen atoms were refined anisotropically and H atoms were visible in difference maps but placed in idealized positions in the refinements. For Hood9, both the BF_4^- anion and the solvent molecule were disordered into two orientations, and restraints were necessary to model the disorder. Crystal data, data collection and refinement parameters are given in Table S1, and the results have been deposited in CIF format at the Cambridge Crystallographic Data Centre.

Table S1. Crystal Data and Structure Refinement

Compound	$[Co(CO)_3(DPPBz)](BF_4)$ •(2-Me-THF)	$[Co(CO)(DEPBz)_2](BF_4)$	$[Co(CO)(dppe)_2](BF_4)$ •(toluene)
CCDC depos. number	2250984	2250985	2250986
Formula	$C_{33}H_{24}CoO_3P_2 \cdot C_5H_{10}O \cdot BF_4$	$C_{29}H_{48}CoOP_4 \cdot BF_4$	$C_{53}H_{48}CoOP_4 \cdot C_7H_8 \cdot BF_4$
Formula weight	762.33	682.30	1062.66
Crystal system	Monoclinic	Orthorhombic	Monoclinic
Space group	$P2_1/n$	Pbcm	$P2_1/n$
a/Å	9.4434 (9)	10.4488 (3)	12.3631 (13)
b/Å	17.8205 (18)	17.1718 (4)	15.1726 (14)
c/Å	21.238 (2)	17.9669 (4)	27.817 (3)
β/deg	102.601 (6)	90	99.066 (7)
V/Å^3	3488.0 (6)	3223.71 (14)	5152.7 (9)
Z	4	4	4
T/K	90.0(5)	100.0(5)	90.0(5)
D_{calc}/g cm^{-3}	1.452	1.406	1.370
Cryst dimen/mm	0.12 × 0.09 × 0.06	0.33 × 0.32 × 0.05	0.17 × 0.08 × 0.01
θ limits, deg	1.5 – 30.6	2.0 – 33.8	1.5 – 28.4
Reflns, measd/unique/obsd	57313 / 10574 / 6629	64938 / 6641 /5296	83914 / 12830 / 8046
Data / params / restraints	10574/ 495 / 106	6641 / 190 / 0	12830 / 641 / 0
F(000)	1568	1432	2208
Radiation	Mo $K\alpha$	Mo $K\alpha$	Mo $K\alpha$
μ/mm^{-1}	0.65	0.78	0.51
R_{int}	0.102	0.053	0.129
$R[I > 2\sigma(I)]$	0.056	0.030	0.048
R_w (all data)	0.131	0.074	0.099
GOF	1.01	1.03	1.00
$\Delta\rho_{max}/\Delta\rho_{min}$, e Å^{-3}	1.23, -0.66	0.58, -0.39	0.46, -0.37

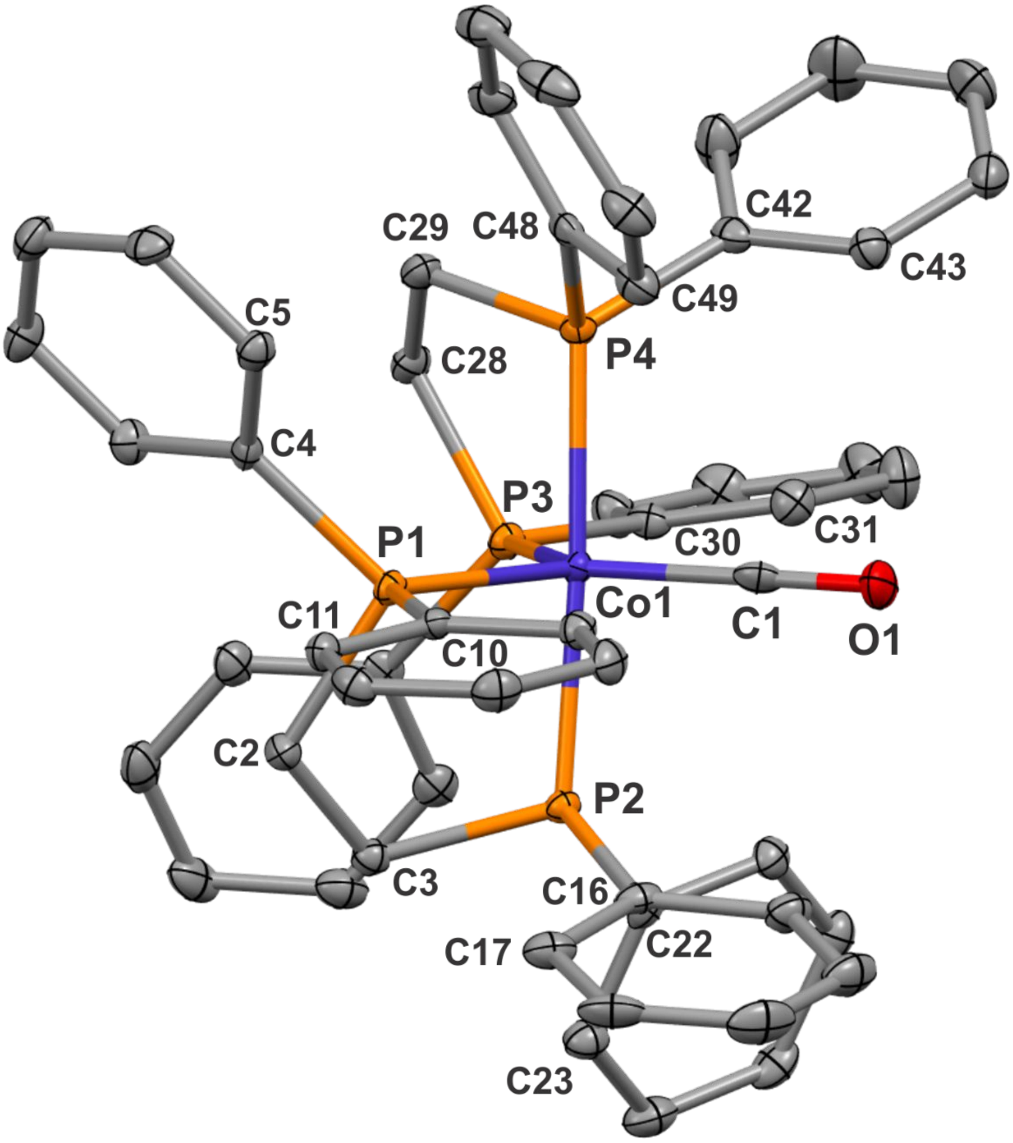

Figure S18. Thermal ellipsoid plot of [Co(CO)(dppe)$_2$](BF$_4$)•(toluene). Hydrogen atoms, BF_4^-, and toluene solvent not shown. Key bond distances (Å) and angles (°): Co1-C1 = 1.747, C1-O1 = 1.160, Co1-P1 = 2.237, Co1-P2 = 2.208, Co1-P3 = 2.238, Co1-P4 = 2.200, P1-Co1-P3 = 112.49, P1-Co1-C1 = 123.53, P3-Co1-C1 = 123.95, Co1-C1-O1 = 179.60, P2-Co1-P4 = 176.59, P1-Co1-P2 = 82.08, P3-Co1-P4 = 82.81, P2-Co1-C1 = 90.22, P4-Co1-C1 = 93.16.

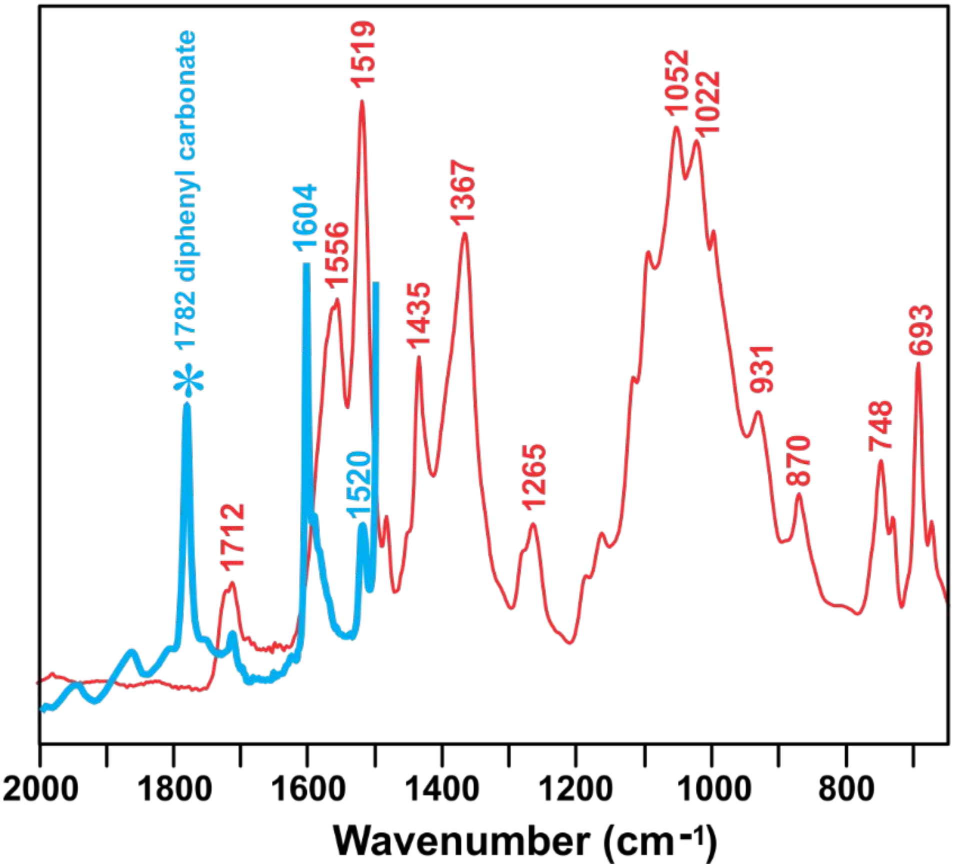

Figure S19. FT-IR comparison of [Co(acac)(DPPBz)](BF_4) catalyst precursors. Blue = material reported by Franke & Zhang,[2] Red = material reported by Stanley & coworkers.[1] The IR spectrum reported by Franke & Zhang was run in solution with some diphenyl carbonate added as a reference and only the small portion shown was reported. Most notably they have a band at 1604 cm^{-1} not present in our sample. Nor do they have a strong band present at 1556 cm^{-1}, which is present in our authentic sample. Their band at 1520 cm^{-1} is far weaker than the band we report at 1519 cm^{-1}.

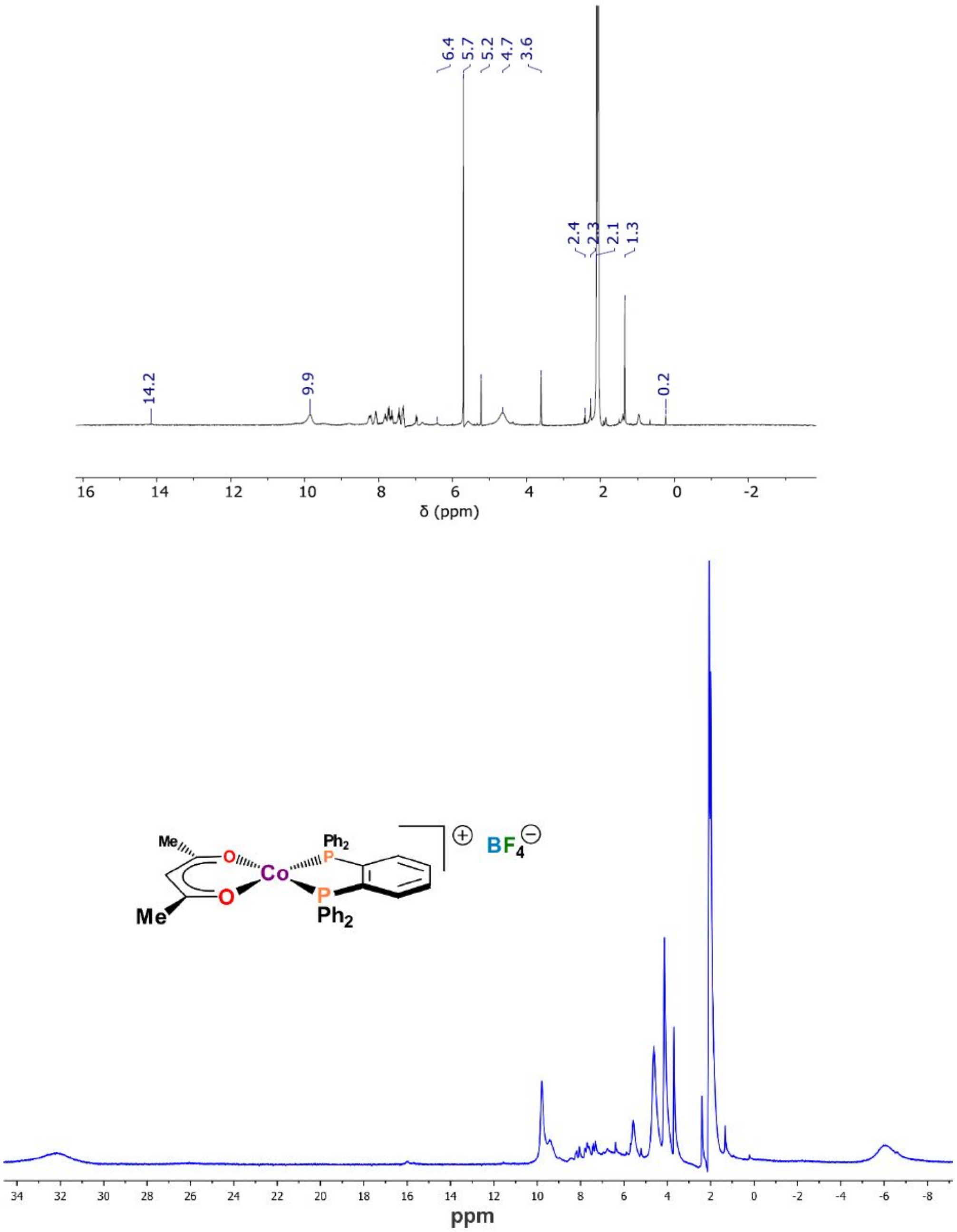

Figure S20. ^{1}H NMR comparison of [Co(acac)(DPPBz)](BF_4) catalyst precursors. Top: ^{1}H NMR is from Franke & Zhang's supplemental material. Bottom: ^{1}H NMR from our work (acetone is contributing to the acac methyl peak at 1.8 ppm along with some dioxane solvent at 3.5 ppm). Note the presence of broadened bands around 32 and –6 ppm in our sample NOT present in their ^{1}H NMR spectrum. Additionally, they report broad bands in the ^{31}P NMR spectrum at 54.8 and 73.6 ppm that they assign to [Co(acac)(DPPBz)](BF_4), along with a presumed impurity peak at 12.4 ppm. We do not observe any ^{31}P NMR bands for [Co(acac)(DPPBz)](BF_4), as one might expect, for phosphine ligands directly bound to a paramagnetic Co(II) center. Reprinted with permission from Refs. 1 & 2.

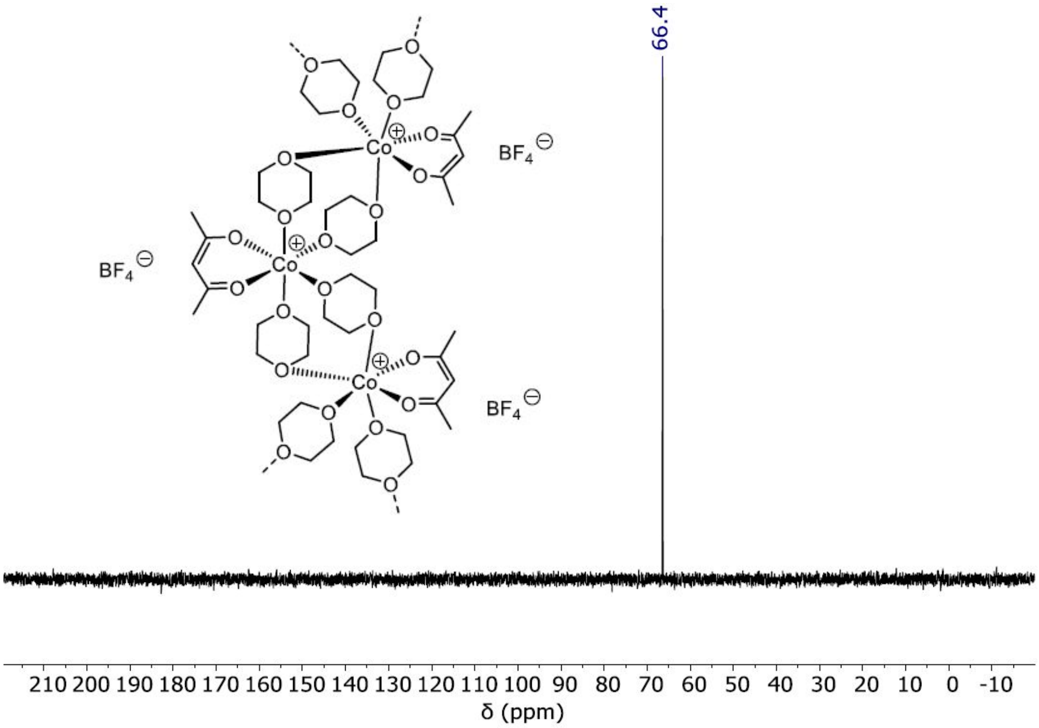

Fig. S2. ^{13}C NMR spectrum of Co1 in D_2O. Only carbons of 1,4-dioxane are detectable at 66.4 ppm due to paramagnetic cobalt(II).

Figure S21. Proposed structure of $[Co_m(acac)_m(\mu\text{-dioxane})_n](BF_4)_m$ and ^{13}C NMR by Franke & Zhang. They reported two versions of this material, one with 57 dioxane solvent molecules per cobalt (labeled **Co1**) and the other with 31 dioxanes per cobalt (labeled **Co5**). They used the 31 dioxane **Co5** material to prepare the $[Co(acac)(bisphosphine)](BF_4)$ catalyst precursors used in their hydroformylation studies. The large excess of dioxane present in both materials casts doubt on the proposed structures with bridging dioxanes between the cobalt centers and on the nature of the material used to prepare the bisphosphine catalyst precursors. We report ^{13}C NMR resonances for _both_ the acac ligand and dioxane at 208.6 ($\Delta v_{1/2}$ = 5.7 Hz), 66.2 ($\Delta v_{1/2}$ = 2.8 Hz), and 30.4 ($\Delta v_{1/2}$ = 3.7 Hz) ppm in the $[Co(acac)(dioxane)_4](BF_4)$, starting material used to make the bisphosphine catalyst precursors. Reprinted with permission from Ref. 2.

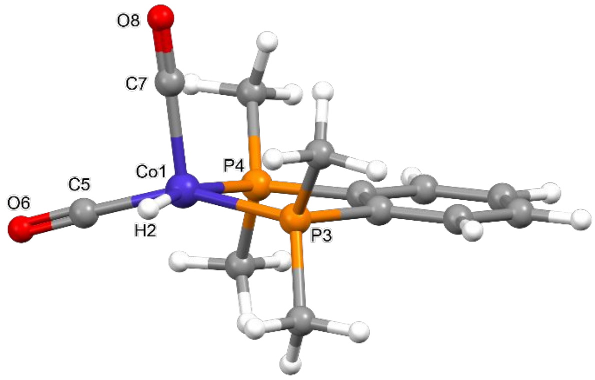

Low-Spin Model

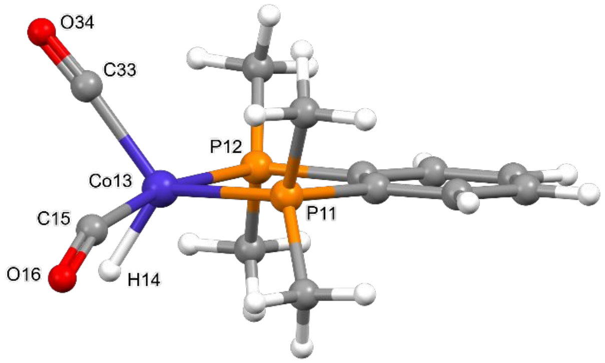

High-Spin Model

ν_{CO} (exp) = 2028, 1989 cm^{-1}

Low-Spin Model

ν_{CO} (DFT) = 2105, 2076 cm^{-1}

Co1-H2 = 1.483 Å
Co1-P3 = 2.242 Å
Co1-P4 = 2.280 Å
Co1-C5 = 1.819 Å
Co1-C7 = 1.871 Å
C5-O6 = 1.133 Å
C7-O8 = 1.133 Å

P3-Co1-P4 = 88.40°
P3-Co1-H2 = 80.73°
P4-Co1-H2 = 166.64°
P3-Co1-C5 = 153.61°
P3-Co1-C7 = 101.92°
P4-Co1-C7 = 101.38°
H2-Co1-C5 = 84.55°
H2-Co1-C7 = 88.52°
C5-Co1-C7 = 99.45°
Co1-C5-O6 = 175.65°
Co1-C7-O7 = 173.57°

High-Spin Model

ν_{CO} (DFT) = 2140, 2076 cm^{-1}

Co13-H14 = 1.572 Å
Co13-P11 = 2.435 Å
Co13-P12 = 2.458 Å
Co13-C15 = 2.135 Å
Co13 – C33 = 2.015 Å
C15-O16 = 1.132 Å
C33-O34 = 1.125 Å

P11-Co13-P12 = 85.50°
P11-Co13-H14 = 120.61°
P12-Co13-H14 = 105.81°
P11-Co13-C15 = 87.73°
P11-Co13-C33 = 122.05°
P12-Co13-C15 = 166.22°
H15-Co13-C15 = 69.62°
H15-Co13-C33 = 111.38°
C15-Co13-C33 = 87.70°
Co13-C15-O16 = 155.13°
Co13-C33-O34 = 174.77°

Figure S22. Gaussian 16 DFT calculated structures for $[HCo(CO)_2(DMPBz)]^+$ using low-spin and high-spin models. DMPBz = $(Me_2P)_2$-1,2-C_6H_4. B3LYP functional using all electron 6-311G(d,p) basis sets for both calculations. The structure for the low-spin model is closest to square-pyramidal, while the structure of the high-spin model is considerably more distorted and closer to trigonal bipyramidal. Frequency calculations showed all positive vibrations. Calculated ν_{CO} frequencies scaled by 0.966.

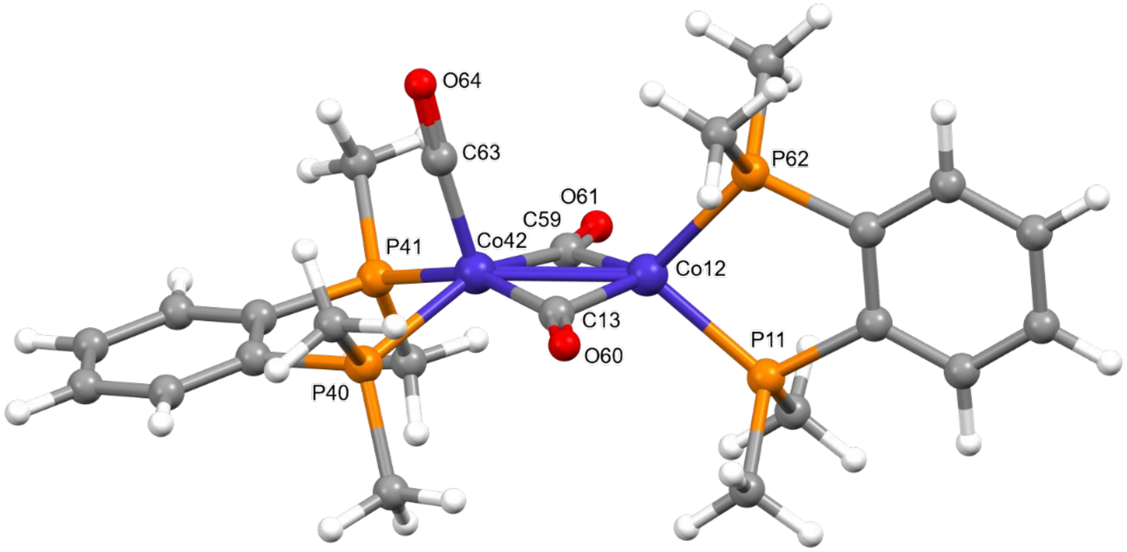

Selected bond distances (Å) and angles (°):

Co42-Co12	2.414		P40-Co42-P41	85.36
Co42-P41	2.281		P40-Co42-C63	98.80
Co42-P40	2.283		P41-Co42-C63	97.24
Co42-C59	1.840		P40-Co42-C13	84.55
Co42-C13	1.892		P42-Co42-C59	83.70
Co42-C63	1.791		Co12-Co42-C63	105.35
C59-O61	1.169		C63-Co42-C13	101.09
C13-O60	1.172		Co42-C63-O64	176.98
C63-O64	1.136		Co42-C13-O60	148.40
Co12-P11	2.213		Co42-C59-O61	155.60
Co12-P62	2.239		P11-C12-P62	87.56
Co12-C59	1.880		Co42-Co12-P11	139.02
Co12-C13	1.825		Co42-Co12-P62	132.31

CO stretching frequencies (cm^{-1}):

Terminal CO:	2025 (exp, estimated)	2069 (DFT, intensity = 512)
Bridging CO:	1888 (exp)	1827 (DFT, intensity = 603)
		1856 (DFT, intensity = 98)

Figure S23. Gaussian 16 DFT Calculation on $[Co_2(\mu\text{-}CO)_2(CO)(DMPBz)_2]^{2+}$
B3LYP functional and 6-311G(d,p) full basis set used on all atoms. Frequency calculation showed all positive vibrations. Calculated ν_{CO} frequencies scaled by 0.966.

References

(1) Hood, D. M.; Johnson, R. A.; Carpenter, A. E.; Younker, J. M.; Vinyard, D. J.; Stanley, G. G. Highly active cationic cobalt(II) hydroformylation catalysts. *Science* **2020**, *367*, 542-548. DOI:10.1126/science.aaw7742

(2) Zhang, B.; Kubis, C.; Franke, R. Hydroformylation catalyzed by unmodified cobalt carbonyl under mild conditions. *Science*, **2022**, *377*, 1223-1227. DOI: 10.1126/science.abm4465
